# Supplementary material for: Protocol for Rhapsody: a longitudinal observational study examining the feasibility of speech phenotyping for remote assessment of neurodegenerative and psychiatric disorders
Source: BMJ Open. 2022 Jun 6;12(6):e061193. doi: 10.1136/bmjopen-2022-061193 (PMC9171270; doi:10.1136/bmjopen-2022-061193)
Supplement: Author's manuscript [file bmjopen-2022-061193.draft_revisions.pdf]

BMJ Open

BMJ Open is committed to open peer review. As part of this commitment we make the peer review history of every article we publish publicly available.

When an article is published we post the peer reviewers' comments and the authors' responses online. We also post the versions of the paper that were used during peer review. These are the versions that the peer review comments apply to.

The versions of the paper that follow are the versions that were submitted during the peer review process. They are not the versions of record or the final published versions. They should not be cited or distributed as the published version of this manuscript.

BMJ Open is an open access journal and the full, final, typeset and author-corrected version of record of the manuscript is available on our site with no access controls, subscription charges or pay-per-view fees (http://bmjopen.bmj.com).

If you have any questions on BMJ Open's open peer review process please email info.bmjopen@bmj.com

BMJ Open

Protocol for RHAPSODY: a longitudinal observational study examining the feasibility of speech phenotyping for remote assessment of neurodegenerative and psychiatric disorders

Journal:	*BMJ Open*
Manuscript ID	bmjopen-2022-061193
Article Type:	Protocol
Date Submitted by the Author:	19-Jan-2022

Complete List of Authors:	Hampsey, Elliot; King's College London, Institute of Psychiatry, Psychology, & Neuroscience<br>Meszaros, Marton; Novoic Limited<br>Skirrow, Caroline; Novoic Limited<br>Strawbridge, Rebecca; King's College London, Institute of Psychiatry, Psychology, & Neuroscience<br>Taylor, Rosie H; King's College London, Institute of Psychiatry, Psychology, & Neuroscience<br>Chok, Lazarus; Novoic Limited<br>Aarsland, D; King's College London, Institute of Psychiatry, Psychology, & Neuroscience<br>Al-Chalabi, Ammar; King's College London, Institute of Psychiatry, Psychology, & Neuroscience<br>Chaudhuri, Ray; King's College London, Institute of Psychiatry, Psychology & Neuroscience; King's College Hospital NHS Foundation Trust, Parkinson's Foundation Centre of Excellence<br>Weston, Jack; Novoic Limited<br>Fristed, Emil; Novoic Limited<br>Podlewska, Aleksandra; King's College London, Institute of Psychiatry, Psychology, & Neuroscience; King's College Hospital NHS Foundation Trust, Parkinson's Foundation Centre of Excellence<br>Awogbemila, Olabisi; King's College Hospital NHS Foundation Trust, Parkinson's Foundation Centre of Excellence<br>Young, Allan; King's College London, Institute of Psychiatry, Psychology, & Neuroscience

Keywords:	Dementia < NEUROLOGY, Depression & mood disorders < PSYCHIATRY, Motor neurone disease < NEUROLOGY, Parkinson-s disease < NEUROLOGY

SCHOLARONE™
Manuscripts

Title: Protocol for RHAPSODY: a longitudinal observational study examining the feasibility of speech phenotyping for remote assessment of neurodegenerative and psychiatric disorders

Elliot Hampsey[1], Marton Meszaros*[2], Caroline Skirrow[2], Rebecca Strawbridge[1], Rosie H Taylor[1], Lazarus Chok[2], Dag Aarsland[1], Ammar Al-Chalabi[1], K Ray Chaudhuri[1, 3], Jack Weston[2], Emil Fristed[2], Aleksandra Podlewska[1, 3], Olabisi Awogbemila[3], Allan H Young[1].*

* EH & MM contributed equally to this manuscript

[1] Institute of Psychiatry, Psychology, & Neuroscience, King's College London, London, UK

[2] Novoic Limited, London, UK

[3] Parkinson's Foundation Centre of Excellence at King's College Hospital NHS Foundation Trust, London, UK

Corresponding author: Mr Elliot Hampsey; elliot.hampsey@kcl.ac.uk

Abstract

Introduction: Neurodegenerative and psychiatric disorders (NPDs) confer a huge health burden, which is set to increase as populations age. New, remotely delivered diagnostic assessments that can detect early-stage NPDs by profiling speech could enable earlier intervention and fewer missed diagnoses. The feasibility of collecting speech data remotely in those with NPDs should be established.

Methods & analysis: The present study will assess the feasibility of obtaining speech data, collected remotely using a smartphone app, from individuals across three NPD cohorts; neurodegenerative cognitive diseases (n=50), other neurodegenerative diseases (n=50), and affective disorders (n=50), in addition to matched controls (n=75). Participants will complete audio-recorded speech tasks and both general and cohort-specific symptom scales. Participants will then remotely self-administer speech tasks and follow-up symptom scales over a 4-week period. The primary objective is to assess the feasibility of remote collection of continuous narrative speech across a wide range of NPDs using self-administered speech tasks. Additionally, the study evaluates if acoustic and linguistic patterns can predict diagnostic group, as measured by the sensitivity, specificity, Cohen's kappa, and area under the receiver operating characteristic curve of the binary classifiers distinguishing each diagnostic group from each other.

Ethics & dissemination: The study received ethical approval from the HRA and Health and Care Research Wales (REC reference: 21/PR/0070). Results will be disseminated through open access publication in academic journals, relevant conferences, and other publicly accessible channels. Results will be made available to participants upon request.

Article Summary:

Trial registration number: NCT04939818; Pre-results.

Keywords: Dementia; Depression & Mood Disorders; Motor Neurone Disease; Parkinson's Disease

Word count: 3723

Strengths & limitations of this study:

- Remote, partially self-administered speech-battery enabling use by those with difficulty accessing in-person healthcare
- Speech battery and study design uniquely allows for investigation of the same tasks across a wide number of indications
- Repeated assessment allows for examination of test reliability, and intra- and inter- subject variability across key clinical groups
- Limited sample size
- Short follow-up period limits scope for longitudinal disease monitoring

For peer review only

Introduction

'Big data' and artificial intelligence approaches have the potential to provide unique and innovative solutions for reducing disease burden. Herewith, we describe the research protocol of the "Rhapsody" study, funded by the National Institute for Health Research, which aims to assess the feasibility of recording and detecting changes in patterns of speech across a range of high burden neurological and psychiatric disorders (NPDs).

<u>Healthcare costs of neurological and psychiatric disorders</u>

Neurological and psychiatric disorders affect 20% of older people[1], at an estimated cost to the UK of >£68b annually[2-5]. With over 65's estimated to make up over a quarter of the UK population by 2050[6], the burden of NPDs on society will increase dramatically. Alzheimer's Disease accounts for 50-70% of dementia cases, with an estimated £34.7b annual healthcare cost in the UK[2]. Dementia with Lewy Bodies (DLB) accounts for 15-20% of all dementia cases[7-9], affecting >100,000 people in the UK. DLB is a much more rapidly progressing disease, with a median survival of 3.72 years post diagnosis[10].

Whilst less prevalent than dementias, Parkinson's disease and Motor Neurone Disease (MND) confer extensive costs due to high morbidity and mortality. Parkinson's disease affects >160,000 people in the UK[11], costing the NHS >£2.2b annually[12], and over 80% may progress to dementia in the long term while the rate is higher in those with the cholinergic subtype[13]. MND affects about 5,000 people in the UK[14] with patients suffering severe impairments in addition to considerable lifespan reduction[15].

Although the average age at onset for affective disorders is typically late 20's to early 30's[16], affective disorders are also marked by recurrence throughout the lifespan[17]. The World Health Organisation reports major depressive disorder (MDD) to have the highest disability burden of all conditions internationally[18], incurring a care cost in the UK of >£23.8b annually[4]. Although less prevalent (1-4.5% lifetime prevalence[19,20]), the burden of bipolar disorder remains considerable, costing the NHS £1.6b annually[5] due partly to the recurrent and chronic disability experienced both within and between mood episodes. People with bipolar disorder have an average of 10-20 years reduced lifespan compared to the general population, due in part to the 20% of people with bipolar disorder who die by suicide[21]. In the UK, bipolar-related unemployment and suicide are associated with £1.5b and £179 million costs respectively[19].

<u>Diagnostic challenges</u>

NPDs are complicated by challenges to accurate and early diagnosis. Estimates suggest that approximately 50% of depressive episodes go undetected[22], whilst >60% of MDD and >90% of bipolar disorder patients may be misdiagnosed[23]. Similarly, ≥50% of those who show evidence of DLB post mortem were not diagnosed with the disease during life[24], with one study finding that 39.5% of those diagnosed clinically as free from Alzheimer's disease met the minimum histopathological threshold[25]. High rates of diagnostic errors in bipolar disorder lead to the delaying of a correct diagnosis by 5.7 years[26], with a substantial proportion waiting for more than a decade[27]. Ostensibly, mis/delayed diagnosis impairs treatment provision, potentially exacerbating course of illness, limiting effective treatment options, and reducing quality of life[28].

Overlap between the clinical presentation of NPDs at the point-of-care makes diagnosis challenging. For example, cognitive impairment , the hallmark symptom of prodromal AD, is also seen in all DLB patients[29] as well as in >80% of Parkinson's disease patients across all motor stages of the condition[30] and is common in people with depression. The cognitive assessments used to screen for dementia are inadequate, leaving 32% of early-stage AD[31] and 50% of DLB patients[32] undiagnosed. Diagnosing other neurodegenerative disorders such as Parkinson's

For peer review only - http://bmjopen.bmj.com/site/about/guidelines.xhtml

disease is particularly challenging, as there are no definitive tests[33]. Physicians instead rely on clinical judgement[34] which is prone to error, with an estimated 20% of those with Parkinson's disease who have come to medical attention going undiagnosed[35]. Similarly, bipolar disorder is commonly misdiagnosed as MDD, with no distinguishing factors between the two illnesses unless manic symptoms are adequately assessed.

Even when diagnoses are made correctly, this is often months if not years after symptoms have begun. Failure to intervene early in disease course is associated with a more severe impact on factors effecting quality of life, such as memory problems[36], motor impairment[37], and psychiatric symptoms such as depression and anxiety[38]. This is particularly true for terminal diseases such as MND, where delayed diagnosis leads to vastly reduced quality of life in remaining years[39].

Novel strategies to improve illness recognition

Artificial intelligence-based techniques could potentially detect subtle and early changes in speech and translate them into predictive diagnostic models. This would assist care providers and their patients, who are likely to benefit from objective profiling biomarkers that are capable of disambiguating clinically similar NPDs. Speech/language alterations in NPDs are promising universal biomarkers as they reflect subtle cognitive, motor and mood changes[40,41]. As speech is among the first modalities to be affected in NPDs[41-44], developing speech/language-based profiling biomarkers will also aid in early diagnosis of typically late-presenting NPDs, like Alzheimer's disease and Parkinson's disease.

Study objectives

The primary objective of the study is to evaluate the feasibility of eliciting continuous narrative speech to collect speech data remotely in three groups of NPDs: neurodegenerative cognitive disorders, other neurodegenerative disorders (Parkinson's disease and MND), and affective disorders. We hypothesise that due to the simple speech-based interface, participants will be able to engage with and provide speech data during virtual study visits and remote assessments. This will be measured by the average length of speech elicitation for each speech task (in seconds) during the first week of self-assessment. Additionally, we will examine the proportion of participants in whom narrative speech lasting at least 20 seconds is elicited within each group.

The study also has secondary objectives concerning the feasibility of using speech tasks to collect speech data in the remote setting, such as evaluating reliability of repeated assessments across related (comparing virtual visits vs. fully remote assessments) or repeated tasks (parallel variants administered across days), examining intra- and inter-individual variance. We will also evaluate adherence to daily remote assessments, and participant-rated usability of the remotely administered application, measured via a brief usability questionnaire completed on the app.

Further, the study will examine whether acoustic and linguistic patterns can be used to distinguish from group-specific control participants, and from other clinical indications, and whether this is impacted by relevant contextual or disease information covariates.

Methods and analysis

Design

The Rhapsody study is an observational longitudinal study during which participants remotely complete a set of tasks designed to elicit speech at baseline and during the 4-week follow-up period. Participant-rated questionnaires of clinical symptoms are completed at baseline and repeated at weeks 2 and 4 of follow-up.

Participants

For peer review only

Potential participants will be identified through local clinical services, cohort-specific internal databases, advertisements on a variety of social media platforms, and dedicated websites for research participation. Participant recruitment will continue from July 2021 to July 2022.

Participants will meet group-specific eligibility criteria for neurodegenerative cognitive disorders (n=50), or other neurodegenerative motor disorders (n=50), or affective disorders (n=50), in addition to matched controls (n=75). The sample size was based on a review of feasibility study group sizes[45]. Individuals must be native English speakers, have access to a smartphone and personal computer/laptop which can connect to the internet and is capable of audio recording as well as running an operating system of macOS X with macOS 10.9 or later; or Windows 7 or above. Exclusion criteria include: current substance use disorder, recent history of stroke (within the last 2 years), recent history of transient ischemic attack or unexplained loss of consciousness (within 12 months) or current risk of suicide. The additional group-specific eligibility criteria are:

Group 1: Neurodegenerative cognitive disorders (n=50): Participants must be aged 50-85, with a score of 20-40 on The Telephone Interview for Cognitive Status – modified[46] (TICS-M) and one of the following clinical diagnoses (within the last 5 years):

A. MCI due to Alzheimer's Disease or mild Alzheimer's dementia as per the National Institute of Aging - Alzheimer's Association core clinical criteria (2011)[47]
B. Probable or possible Dementia with Lewy Bodies as per The Dementia with Lewy Bodies Consortium definition[48].
C. Cognitive Impairment (not due to Alzheimer's/DLB), either diagnosed with behavioural variant fronto temporal dementia[49], semantic variant primary progressive aphasia or nonfluent variant primary progressive aphasia[50], or vascular dementia[51].

Group 2: Other neurodegenerative disorders (n=50):

A. Participants with Motor Neuron Disease must be aged 18-85 and score Stage 3 or less on the King's ALS Staging system[52].
B. Parkinson's disease participants must be within 5 years of a diagnosis of idiopathic Parkinson's Disease according to the UK Brain Bank Criteria, aged between 30 and 85, and have a Hoehn and Yahr stage[53] of $\leq$2.

Group 3: Affective disorders (n=50): Participants with affective disorders must be aged 18-65, in a depressive episode according to DSM-IV criteria as assessed by the Mini international Neuropsychiatric Interview (M.I.N.I) v5.0[54], of at least moderate severity as assessed on the Clinical Global Impressions Scale[55] either in the context of MDD or bipolar disorder (types I or II).

Group 4: Unaffected Controls (n=75)*:* Approximately 25 unaffected control participants will be recruited for each cohort, matched for gender, age and education levels. Participants will be in otherwise good health; they may experience mild disorders (of metabolic, respiratory, immunological, cardiologic, and metabolic origin) that do not impair daily functioning.

<u>Data Collection Procedure</u>

Study procedures are summarised in figure 1. Potential participants will be provided with a patient information sheet by the research team, to be followed by the study visit $\geq$24 hours later. Participants providing consent will complete the study visit via video conferencing software. Participants will first complete the screening and baseline assessment phase. During screening, participant eligibility is evaluated against inclusion/exclusion criteria, and where relevant demographic information, medical history and information on current medications are obtained.

For peer review only

Cohort-specific assessments (see table 1) will also be used to determine eligibility. Ineligible individuals will exit the study at this stage.

[Figure 1 here]

Figure 1. Flow diagram of the participant journey in the Rhapsody study.

Study procedure	Screening/ baseline	Self-assessments			
		Week 1	Week 2	Week 3	Week 4
Medical History, Concomitant Medication Overview and Demographics	X				
Telephone Interview for Cognitive Status – modified (Group 1 only)	X				
The Mini-International Neuropsychiatric Interview [a] (psychiatric diagnostic evaluation) (Group 3 only)	X				
Clinical Global Impression (Group 3 only)	X				
Speech/language assessments	X	X	X	X	X
Inventory of Depressive Symptomatology – Clinician Rated (Group 3 only)	X				
Patient Health Questionnaire – 9 item*	X		X		X
Generalised Anxiety Disorder Assessment – 7 item*	X		X		X
Maudsley 3-Item Depression Visual Analogue Scale*	X		X		X
Altman Self-Rating Mania Scale* (Groups 2 and 3 only)	X		X		X

Table 1. Screening tools and questionnaires completed at baseline and follow-up.
*Patient-rated
[a]Suicide questionnaire administered if participant scores $\geq$10 on the Patient Health Questionnaire – 9 item

A. [All groups]: The Patient Health Questionnaire – 9-item[56] is a short, self-report tool which uses 4-point Likert scales to assess 9 items relating to depressive symptomology.
B. [Group 3]: The Mini-International Neuropsychiatric Interview (M.I.N.I) is a structured interview for mental health diagnoses according to the Diagnostic and Statistical Manual of Mental Disorders criteria. The suicidality scale will be completed by any participant scoring $\geq$10 on the Patient Health Questionnaire – 9-item.
C. [Group 3]: The Clinical Global Impressions Scale is a 3-item, researcher-administered tool that provides an overall ‘global’ judgement concerning the participant’s severity of depressive illness.

D. [Group 1]: The Telephone Interview for Cognitive Status – modified is a short, researcher-administered assessment of cognitive functioning validated for use over the phone.
E. [Group 3]: The Inventory of Depressive Symptomatology (30-item Clinician Rated)[57] is a researcher-rated instrument that provides detailed assessment of depression severity over the previous 7 days.

The participants will then complete the main sequence of speech assessments, in which participants are guided to complete a battery of tasks designed to elicit continuous narrative speech, such as reading text, and describing images. Speech tasks will be administered and recorded using the application, with all participants completing the same set of speech tasks. Participants will be supported with using the Novoic application on their smartphone. Audio recordings will be transferred to a secure server and then deleted from the smartphone device.

Recordings of the combined screening and baseline visit will also be made simultaneously by video conferencing software. Zoom (https://zoom.us) will be used, due to its security features and the ability to turn off audio manipulation effects. The main assessment phase begins with a series of speech tasks, self-recorded using a smartphone application, that the researcher guides the participant to complete. An overview of speech tasks completed in the main assessment is provided in table 2.

**Speech task**	**Task details**
The Automated Story Recall Task with immediate recall	Recordings of narrative stories are played to the participant, which they must recall immediately. The stories are matched for several linguistic and phonological qualities.
Category Fluency Task	Participants are given a category such as 'animals' and required to give as many examples of that category as they can in 60 seconds.
Letter Fluency Task	Participants are given a letter of the alphabet and required to give as many examples of words beginning with that letter as they can in 60 seconds.
Action Fluency Task	Participants are tasked with naming as many of examples of things that "people do", i.e., present tense verbs such as "run" or "work", in 60 seconds.
The Automated Story Recall Task with delayed recall	Participants must recall the narrative stories played to them at the beginning of the speech task phase.
Procedural Discourse Questions	Participants are tasked with describing, in as much detail as possible, how they would go about doing the dishes by hand.
Picture Description Task	Participants verbally describe a singular image in as much detail as they can.
Sequence Narration task	Participants must describe their narrative interpretation of a series of images presented as a storyboard.
Reading a Script	Participants must read a short passage aloud.

For peer review only

Sustained Phonation Task	Participants are required to phonate several sounds such as /a:/ or /u:/ for ten seconds, in one breath.
Syllable Repetition Task	Participants are required to repeatedly phonate a syllable as quickly as possible for ten seconds.
Sentence Repetition task	The researcher will read a sentence aloud to the participant, who must repeat it back exactly.

Table 2. Overview of speech tasks in the main assessment phase.

Following the completion of the main sequence speech tasks, all participants will then be asked to complete online self-rated questionnaires via the Qualtrics platform (www.qualtrics.com)(see table 1):

A. The General Anxiety Disorder Assessment 7-item[58] is a brief tool for assessing symptoms of anxiety. Participants rate 7 items relating to core symptoms of anxiety on a 4-point Likert scale concerning the frequency at which those symptoms occur.

B. The Maudsley 3-Item Depression Visual Analogue Scale[59] is a researcher-rated assessment of depressive illness which detects symptom severity and suicidality.

C. The Altman Self Rating Mania Scale[60] is a 5-item instrument designed to self-assess the presence and severity of manic and hypomanic symptoms by comparing how they feel currently to their non-affected baseline state.

Participants will then complete follow up data collection over a period of 4 weeks using the smartphone application. Participants will be notified via the mobile application to complete 12 brief (approximately 15 minute) unsupervised speech and cognition assessment batteries over the month following the baseline visit. Participants will receive a different set of speech tasks each day from the application's built-in task-bank, including the same main speech and cognition tasks described above, and abbreviated test of executive function (Stroop, digit span).

Additional, brief self-report measures, incorporated into remote assessments, included:

- A 4-item questionnaire on the usability of the smartphone application
- A brief, 3-minute-long questionnaire on whether the participant has any visual or hearing impairments, and whether they had related difficulties with the administered tasks.
- A baseline 4-item questionnaire: examining typical mood, sleep mind wandering and effort
- A brief, daily 4-item questionnaire on current state: mood, sleep mind wandering and effort

The remote assessment schedule is provided in table 3. Participants will also complete follow-up questionnaires online via Qualtrics during weeks 2 and 4, which are shown in table 1.

	**Remote assessment day**											
**Task**	**1**	**2**	**3**	**4**	**7**	**8**	**9**	**10**	**11**	**14**	**21**	**28**
Counting 1-10 or 10-1	X	X	X	X	X	X	X	X	X	X	X	X
Automated Story Recall Task immediate recall tasks	X	X	X	X	X	X	X	X	X	X	X	X
Category fluency	X					X				X	X	X
Letter fluency		X					X			X	X	X

For peer review only

Action fluency										X		
Digit span forward			X					X				
Digit span backward			X					X				
Stroop					X							
Procedural discourse				X					X			
Picture description		X					X					
Sustained phonation										X		
Syllable repetition	X									X		
Sentence repetition	X									X		
Automated Story Recall Task delayed recall tasks	X	X	X	X	X	X	X	X	X	X	X	X
Reading a script												X
Usability questionnaire		X							X			
Vision and hearing questionnaire			X									
Baseline mood, sleep, attention & effort questionnaire	X											
Daily mood, sleep, attention & effort questionnaire	X	X	X	X	X	X	X	X	X	X	X	X

Table 3. Assessments completed remotely via the application on the participants' own smartphones. Remote assessment day corresponds to the number of days after the virtual visit. Crosses correspond to scheduled assessments during these days, and test order is shown in descending order. As such, all Automated Story Recall Task immediate and delayed recall assessments are completed with distractor tasks occurring in-between.

For peer review only

Statistical analysis plan

The main analysis objectives of the study include examining: (1) feasibility of eliciting and collecting continuous narrative speech within different clinical groups. (2) reliability, and intra and inter-subject variance of speech task performance, (3) adherence to remote unsupervised assessments, (4) app usability, and (5) to evaluate whether acoustic and linguistic patterns can be used to distinguish clinical groups from controls, and between clinical indications. More detailed analysis plans for meeting these objectives are described below:

Feasibility will be assessed as the length of speech generated during speech elicitation tasks in virtual visits and remotely. The primary endpoint is calculated as the number of participants who successfully complete Automated Story Recall Task assessments during the main assessment phase as a fraction of the disease cohort. Participants are considered successful where at least one of the immediate story recalls produces a spoken response spanning ≥20 seconds from the first to the last word. The proportion of participants able to elicit significant speech in each group will be used to indicate likely suitability of this assessment procedure within each disease group.

Reliability will be measured by examining the stability of equivalent tasks completed over the assessment period. This will include examining reliability across parallel task variants (administered over different assessment days during remote assessment), and comparison of equivalent test variants in different settings (virtual study visit vs. remote assessment).

Correlational analysis will be completed between parallel test variants, and intraclass correlations will be carried out to examine test-retest reliability of the same test variants administered across days and between settings (virtual study visit vs. remote assessment). Coefficients of individual agreement will be used as a measure of inter-participant and intra-participant variability.

Usability will be examined via the average total score on the usability questionnaire for each disease cohort and their matched controls, to examine disease cohort-specific problems or difficulties in completing remote assessments. Adherence will be defined as the proportion of participants engaging in daily remote assessments in each indication. Data will be analysed using t-tests, ANOVAs or non-parametric equivalents, as appropriate.

For the endpoints which require training classifiers or regressors to make predictions, the entire data set will be used for both training machine learning models and validating their statistical properties. Machine learning methods used may include supervised, unsupervised, semi-supervised and reinforcement learning methods for forming intermediate representations of the data and performing the final classification/regression analyses.

Speech measures will be derived using several methods from the fields of signal/speech processing and natural language processing to investigate which speech markers are most predictive of clinical status. Deep learning methods will be utilised, where the speech measures are derived as internal representations of the models. Methods used here will include unsupervised context-aware word embeddings, a way of distinguishing between identical words with different contextual meanings, and audio representation learning.

Training and validation of the ML models will be performed using cross-validation techniques. Performance of classification analysis will be characterised by the area under the receiver operating characteristic (ROC) curve (AUC), sensitivity, specificity, and Cohen's kappa. Demonstration that a binary classifier performs better than the random baseline will be done via mapping AUC estimates to a p-value via the Mann-Whitney U statistic[61].

Methodological issues

The primary potential limitation of the study is poor compliance with the self-administered speech tasks over the follow-up period which could limit the amount of data available for comparing acoustic and linguistic features between groups. Efforts to address this potential issue include reminder notifications from the smartphone application and allowing participants to complete these self-assessments at a time to suit them, rather than being scheduled. It should also be noted that adherence to the self-administered tasks is itself an outcome measure of the study, and so missing data in this regard is informative.

Data management and oversight:

All aspects of the study will be overseen by the Study Management Group. Primary investigators will oversee and manage all aspects of their study in their respective cohorts. Their responsibility includes monitoring and ensuring the safety of all participants in their respective cohorts, including the reporting of any Adverse or Serious Adverse events, and managing participant withdrawal/discontinuation.

Audio data will be recorded by the app and by the video conference software. Upon completion of each assessment, the audio will be securely transferred to Novoic's secure server. Novoic's infrastructure has been built in compliance with the General Data Protection Regulation (GDPR). Other clinical data will be stored in a secure password-protected server, compliant with

For peer review only - http://bmjopen.bmj.com/site/about/guidelines.xhtml

all applicable laws and regulations, including ICH E6 Good Clinical Practice (GCP), EU Annex 11, GDPR.

Data Access
The speech data obtained during the study will not be made available to a repository due to the potential for participant identification.

Ethics and dissemination
The study received REC approval from the London - Queen Square Research Ethics Committee on the 15th April 2021, and HRA approval from HRA and Health and Care Research Wales (HCRW) on the 23rd April 2021. (REC reference: 21/PR/0070). Findings will be published in peer-reviewed journals and presented at conferences focussed on neuroscience & psychiatry, and machine learning & natural language processing. Novoic will create a project webpage containing key findings, published papers, and open-source software packages.

Protocol and registration
The study is registered with Clinicaltrials.gov (NCT04939818).

Patient & public involvement
We have involved patients and members of the public in planning of this study, having sought service users' perspectives on protocol documentation which resulted in amendments to language used and patient facing documentation. We have also implemented changes based on carers' feedback from a focus group of 10-15 individuals. We also actively collect usability feedback from service users. During dissemination, we will invite service users and carers to contribute to a public perspective on the interpretation of trial findings.

Author Contributions
Mr Elliot Hampsey (ORCID ID: 0000-0001-6985-5646): contributed to the writing of the study protocol, in addition to the writing and editing of the manuscript for journal submission.
Dr Marton Meszaros (ORCID ID: 0000-0003-4937-3062): main contributor on designing the study and protocol. Contributed to writing the full study protocol and editing the manuscript for journal submission.
Dr Caroline Skirrow (ORCID ID: 0000-0001-8692-7787): contributed to the study design, writing, and editing of the manuscript for journal submission.
Dr Rebecca Strawbridge (ORCID ID: 0000-0002-2984-1124): contributed to the editing of the study protocol and manuscript for journal submission, in addition to study set up activities.
Miss Rosie Hannah Taylor (ORCID ID: 0000-0001-6742-4842): Contributed to editing of the manuscript for journal submission.
Mr Lazarus Chok (ORCID ID: 0000-0002-9514-364X): contributed to the writing of the study protocol, in addition to the editing of the manuscript for journal submission.
Professor Dag Aarsland (ORCID ID: 0000-0001-6314-216X): Contributed to study and protocol design. Principal investigator for neurodegenerative disease cohort.
Professor Ammar Al-Chalabi (ORCID ID: 0000-0002-4924-7712): Contributed to study and protocol design. Principal investigator for motor disorders cohort.
Professor K Ray Chaudhuri (ORCID ID: 0000-0003-2815-0505): Contributed to study and protocol design. Principal investigator for motor disorders cohort.
Dr Jack Weston (ORCID ID: 0000-0001-5344-7840): contributed to the writing of the study protocol, in addition to editing of the manuscript for journal submission.
Mr Emil Fristed (ORCID ID: 0000-0002-9590-7275): main contributor on designing the study and protocol. Contributed to writing the full study protocol and editing the manuscript for journal submission.

For peer review only

Ms Aleksandra Podlewska (ORCID ID: 0000-0002-0805-3356): Contributed to editing of the manuscript for journal submission.
Ms Olabisi Awogbemila (ORCID ID: 0000- 0002- 6814- 3097): Contributed to editing of the manuscript for journal submission.
Professor Allan H. Young (ORCID ID: 0000-0003-2291-6952): Chief Investigator. Contributed to study and protocol design and edited the manuscript for journal submission.

Acknowledgements
We would like to thank the NIHR Maudsley Biomedical Research Centre's FAST-R service and Service User Advisory Group. This report is independent research funded by the National Institute for Health Research (Artificial Intelligence, Project Rhapsody: Investigating the clinical feasibility of using AI-based deep audio and language processing techniques to diagnose neurological and psychiatric diseases, AI_AWARD01984) and NHSX. The views expressed in this publication are those of the author(s) and not necessarily those of the National Institute for Health Research, NHSX or the Department of Health and Social Care.

Funding
This work is supported by the National Institute of Health Research. The study has been awarded by the NHS Artificial Intelligence in Health and Care Award (grant number AI_AWARD01984).

Competing interests statement
R.S. has received an honorarium for speaking from Lundbeck. In the past 3 years: A.H.Y. has received honoraria for speaking from AstraZeneca, Lundbeck, Eli Lilly and Sunovion; honoraria for consulting from Allergan, Livanova and Lundbeck, Sunovion and Janssen; and research grant support from Janssen. EH declares no conflicts of interest. EF is CEO of Novoic. MM, JW and CS are employees of Novoic, and EF, MM and JW are shareholders in the company.

References

1. Mental health of older adults [Internet]. Who.int. 2021 [cited 30 September 2021]. Available from: https://www.who.int/news-room/fact-sheets/detail/mental-health-of-older-adults

2. Wittenberg R, Hu B, Barraza-Araiza L, Rehill A. Projections of older people with dementia and costs of dementia care in the United Kingdom, 2019–2040. Care Policy and Evaluation Centre Working Paper 5. 2019.

3. Weir S, Samnaliev M, Kuo T, Tierney T, Walleser Autiero S, Taylor R et al. Short- and long-term cost and utilization of health care resources in Parkinson's disease in the UK. Movement Disorders. 2018;33(6):974-981.

For peer review only

4. No Health Without Mental Health: a cross-government mental health outcomes strategy for people of all ages. 2011.

5. McCrone P, Dhanasiri S, Patel A, Knapp M, Lawton-Smith S. Paying the price: The cost of mental health care in England to 2026. 2008.

6. Overview of the UK population - Office for National Statistics [Internet]. Ons.gov.uk. 2021 [cited 30 September 2021]. Available from: https://www.ons.gov.uk/peoplepopulationandcommunity/populationandmigration/populationestimates/articles/overviewoftheukpopulation/august2019

7. McKeith I, Mintzer J, Aarsland D, Burn D, Chiu H, Cohen-Mansfield J et al. Dementia with Lewy boies. The Lancet Neurology. 2004;3(1):19-28.

8. A Guide to Lewy Body Dementia. The Lewy Body Society, 2019.

9. O'Brien, J., Taylor, J., Thomas, A., Bamford, C., Vale, L., Hill, S., Allan, L., et al. Improving the Diagnosis and Management of Neurodegenerative Dementia of Lewy Body Type in the NHS (DIAMOND-Lewy Programme). NIHR Journals Library Publications https://doi.org/10.17863/CAM.65933

10. McKeith I, Rowan E, Askew K, Naidu A, Allan L, Barnett N et al. More Severe Functional Impairment in Dementia With Lewy Bodies Than Alzheimer Disease Is Related to Extrapyramidal Motor Dysfunction. The American Journal of Geriatric Psychiatry. 2006;14(7):582-588.

11. Gumber, A., Ramaswamy, B., Ibbotson, R., Ismail, M., Thongchundee, O., Harrop, D., Allmark, P., & Rauf, A. (2017). Economic, Social and Financial Cost of Parkinson's on Individuals, Carers and their Families in the UK. Project Report. Centre for Health and Social Care Research, Sheffield Hallam University.

12. McCrone P, Allcock L, Burn D. Predicting the cost of Parkinson's disease. Movement Disorders. 2007;22(6):804-812.

13. Marras C, Chaudhuri KR. Nonmotor features of Parkinson's disease subtypes. Mov Disord. 2016 Aug;31(8):1095-102. doi: 10.1002/mds.26510. Epub 2016 Feb 10. PMID: 26861861

14. Gowland A, Opie-Martin S, Scott K, Jones A, Mehta P, Batts C et al. Predicting the future of ALS: the impact of demographic change and potential new treatments on the prevalence of ALS in the United Kingdom, 2020–2116. Amyotrophic Lateral Sclerosis and Frontotemporal Degeneration. 2019;20(3-4):264-274.

15. Westeneng H, Debray T, Visser A, van Eijk R, Rooney J, Calvo A et al. Prognosis for patients with amyotrophic lateral sclerosis: development and validation of a personalised prediction model. The Lancet Neurology. 2018;17(5):423-433.

16. Solmi M, Radua J, Olivola M, Croce E, Soardo L, Salazar de Pablo G et al. Age at onset of mental disorders worldwide: large-scale meta-analysis of 192 epidemiological studies. Molecular Psychiatry. 2021.

17. Kessing L. Recurrence in affective disorder. British Journal of Psychiatry. 1998;172(1):29-34.

18. James S, Abate D, Abate K, Abay S, Abbafati C, Abbasi N et al. Global, regional, and national incidence, prevalence, and years lived with disability for 354 diseases and injuries for 195 countries and territories, 1990–2017: a systematic analysis for the Global Burden of Disease Study 2017. The Lancet. 2018;392(10159):1789-1858.

19. Fajutrao L, Locklear J, Priaulx J, Heyes A. A systematic review of the evidence of the burden of bipolar disorder in Europe. Clinical Practice and Epidemiology in Mental Health. 2009;5(1):3.

For peer review only

20. Hirschfeld R. Differential diagnosis of bipolar disorder and major depressive disorder. Journal of Affective Disorders. 2014;169:S12-S16.

21. Dome P, Rihmer Z, Gonda X. Suicide Risk in Bipolar Disorder: A Brief Review. Medicina. 2019;55(8):403.

22. Mitchell A, Vaze A, Rao S. Clinical diagnosis of depression in primary care: a meta-analysis. The Lancet. 2009;374(9690):609-619.

23. Vermani M, Marcus M, Katzman M. Rates of Detection of Mood and Anxiety Disorders in Primary Care. The Primary Care Companion for CNS Disorders. 2011.

24. Hohl U, Tiraboschi P, Hansen L, Thal L, Corey-Bloom J. Diagnostic Accuracy of Dementia With Lewy Bodies. Archives of Neurology. 2000;57(3):347.

25. Beach T, Monsell S, Phillips L, Kukull W. Accuracy of the Clinical Diagnosis of Alzheimer Disease at National Institute on Aging Alzheimer Disease Centers, 2005–2010. Journal of Neuropathology & Experimental Neurology. 2012;71(4):266-273.

26. Morselli P, Elgie R. GAMIAN-Europe*/BEAM survey I - global analysis of a patient questionnaire circulated to 3450 members of 12 European advocacy groups operating in the field of mood disorders. Bipolar Disorders. 2003;5(4):265-278.

27. Dagani J, Signorini G, Nielssen O, Bani M, Pastore A, Girolamo G et al. Meta-analysis of the Interval between the Onset and Management of Bipolar Disorder. The Canadian Journal of Psychiatry. 2016;62(4):247-258.

28. Rasmussen J, Langerman H. Alzheimer's Disease – Why We Need Early Diagnosis. Degenerative Neurological and Neuromuscular Disease. 2019; Volume 9:123-130.

29. Goldman J, Williams-Gray C, Barker R, Duda J, Galvin J. The spectrum of cognitive impairment in Lewy body diseases. Movement Disorders. 2014;29(5):608-621.

30. Aarsland, D., Andersen, K., Larsen, J.P., Lolk, A. & Kragh-Sørensen, P. (2003). Prevalence and characteristics of dementia in Parkinson Disease: An 8-year prospective study. Archives of Neurology, 60(3), pp.387-92.

31. NHS Digital (2020). Recorded Dementia Diagnoses January 2020. London: NHS England.

32. Price A, Farooq R, Yuan J, Menon V, Cardinal R, O'Brien J. Mortality in dementia with Lewy bodies compared with Alzheimer's dementia: a retrospective naturalistic cohort study. BMJ Open. 2017;7(11):e017504.

33. Parkinson's Disease: Clinical features and diagnosis. Journal of Neurology, Neurosurgery and Psychiatry, 79(4), pp.368-76.

34. Rizek, P., Kumar, N. & Jog, M.S. (2016). An update on the diagnosis and treatment of Parkinson disease. CMAJ, 188(16), pp.1157-65.

35. Schrag A, Ben-Shlomo Y, Quinn N. How valid is the clinical diagnosis of Parkinson's disease in the community? J Neurol Neurosurg Psychiatry. 2002 Nov;73(5):529-34. doi: 10.1136/jnnp.73.5.529. PMID: 12397145; PMCID: PMC1738115.

36. Sperling, R.A., Dickerson, B.C., Pihlajamaki, M., Vannini, P., LaViolette, P.S., Vitolo, O.V., Hedden, T., Becker, J.A., Rentz, D.M., Selkoe, D.J., & Johnson, K.A. (2010). Functional alterations in memory networks in early Alzheimer's disease. Neuromolecular Medicine, 12(1), pp.27-43.

37. Kulisevsky, J., & Pagonabarraga, J. (2009). Cognitive impairment in Parkinson's disease: tools for diagnosis and assessment. Movement Disorders, 24(8): pp.1103-10.

For peer review only

38. D'Onofrio, G., Sancarlo, D., Panza, F., Copetti, M., Cascavilla, L., Paris, F., Seripa, D., Matera, M.G., Solfrizzi, V., Pellegrini, F., & Pilotto, A. (2012). Neuropsychiatric symptoms and functional status in Alzheimer's disease and vascular dementia patients. Current Alzheimer Research, 9(6), pp.759-71.

39. NICE (2015). Motor Neurone Disease Briefing Paper. London: NICE.

40. Boschi, V., Catricalà, E., Consonni, M., Chesi, C., Moro, A., & Cappa, S.F. (2017). Connected Speech in Neurodegenerative Language Disorders: A Review. Frontiers in psychology, 8, 269.

41. Engelman, M., Agree, E.M., Meoni, L.A. & Klag, M.J. (2010). Propositional density and cognitive function in later life: Findings from the precursors study. J Gerontol., 65, pp.706-11.

42. Riley, K., Snowdon, D.A., Desrosiers, M.F. & Markesbery, W.R. (1996). Early life linguistic ability, late life cognitive function, and neuropathology: findings from the Nun Study. Neurobiology of Aging, 26(3), pp.341-7.

43. Cuetos, F., Arango-Lasprilla, J.C., Uribe, C., Valencia, C. & Lopera, F. (2007). Linguistic changes in verbal expression: A preclinical marker of Alzheimer's Disease. J Int Neuropsychol Soc., 13, pp.433-9.

44. Snowdon, D.A., Kemper, S.J., Mortimer, J.A., Greiner, L.H., Wekstein, D.R. & Markesbery, W.R. (1996). Linguistic ability in early life and cognitive function from the nun and Alzheimer's Disease in late life study. JAMA, 275, pp.528-32.

45. Hertzog M. Considerations in determining sample size for pilot studies. Research in Nursing & Health. 2008;31(2):180-191.

46. Welsh, K. A., Breitner, J. C., & Magruder-Habib, K. M. (1993). Detection of dementia in the elderly using telephone screening of cognitive status. *Neuropsychiatry, Neuropsychology, & Behavioral Neurology, 6*(2), 103–110.

47. Albert M, DeKosky S, Dickson D, Dubois B, Feldman H, Fox N et al. The diagnosis of mild cognitive impairment due to Alzheimer's disease: Recommendations from the National Institute on Aging-Alzheimer's Association workgroups on diagnostic guidelines for Alzheimer's disease. Alzheimer's & Dementia. 2011;7(3):270-279.

48. McKeith I, Boeve B, Dickson D, Halliday G, Taylor J, Weintraub D et al. Diagnosis and management of dementia with Lewy bodies. Neurology. 2017;89(1):88-100.

49. Rascovsky K, Hodges JR, Knopman D, Mendez MF, Kramer JH, Neuhaus J, Miller BL, et al. Sensitivity of revised diagnostic criteria for the behavioural variant of frontotemporal dementia. *Brain.* 2011;134:2456–2477.

50. Mesulam M. Primary progressive aphasia. Annals of Neurology. 2001;49(4):425-432.

51. Roman G, Tatemichi T, Erkinjuntti T, Cummings J, Masdeu J, Garcia J et al. Vascular dementia: Diagnostic criteria for research studies: Report of the NINDS-AIREN International Workshop. Neurology. 1993;43(2):250-250.

52. Roche J, Rojas-Garcia R, Scott K, Scotton W, Ellis C, Burman R et al. A proposed staging system for amyotrophic lateral sclerosis. Brain. 2012;135(3):847-852.

53. Hoehn M, Yahr M. Parkinsonism: onset, progression, and mortality. Neurology. 1967;17(5):427-427.

54. Sheehan D, Lecrubier Y, Sheehan K, Amorin P, Janvas J, Weiler E et al. The Mini-International Neuropsychiatric Interview (M.I.N.I.): the development and validation of a

For peer review only

structured diagnostic psychiatric interview for DSM-IV and ICD-10. Journal of Clinical Psychiatry. 1998;Suppl 20:22-33:34-57.

55. Guy, W. (1976) Clinical Global Impressions, ECDEU Assessment Manual for Psychopharmacology, revised (DHEW Publ. No. ADM 76-338). National Institute of Mental Health, Rockville, 218-222.

56. Kroenke K, Spitzer R, Williams J. The PHQ-9. Journal of General Internal Medicine. 2001;16(9):606-613.

57. Rush A, Carmody T, Reimitz P. The Inventory of Depressive Symptomatology (IDS): Clinician (IDS-C) and Self-Report (IDS-SR) ratings of depressive symptoms. International Journal of Methods in Psychiatric Research. 2000;9(2):45-59.

58. Spitzer R, Kroenke K, Williams J, Löwe B. A Brief Measure for Assessing Generalized Anxiety Disorder. Archives of Internal Medicine. 2006;166(10):1092.

59. Moulton C, Strawbridge R, Tsapekos D, Oprea E, Carter B, Hayes C et al. The Maudsley 3-item Visual Analogue Scale (M3VAS): Validation of a scale measuring core symptoms of depression. Journal of Affective Disorders. 2021;282:280-283.

60. Altman E, Hedeker D, Peterson J, Davis J. The Altman Self-Rating Mania Scale. Biological Psychiatry. 1997;42(10):948-955.

61. Mann H, Whitney D. On a Test of Whether one of Two Random Variables is Stochastically Larger than the Other. The Annals of Mathematical Statistics. 1947;18(1):50-60.

For peer review only

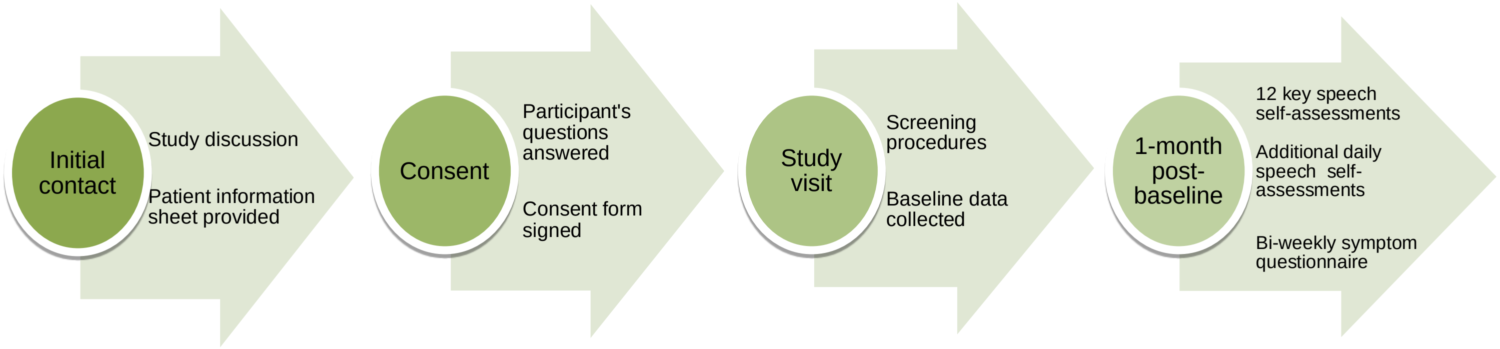

Figure 1.

For peer review only

BMJ Open

Protocol for RHAPSODY: a longitudinal observational study examining the feasibility of speech phenotyping for remote assessment of neurodegenerative and psychiatric disorders

Journal:	*BMJ Open*
Manuscript ID	bmjopen-2022-061193.R1
Article Type:	Protocol
Date Submitted by the Author:	27-Apr-2022

Complete List of Authors:	Hampsey, Elliot; King's College London, Institute of Psychiatry, Psychology, & Neuroscience<br>Meszaros, Marton; Novoic Limited<br>Skirrow, Caroline; Novoic Limited<br>Strawbridge, Rebecca; King's College London, Institute of Psychiatry, Psychology, & Neuroscience<br>Taylor, Rosie H; King's College London, Institute of Psychiatry, Psychology, & Neuroscience<br>Chok, Lazarus; Novoic Limited<br>Aarsland, D; King's College London, Institute of Psychiatry, Psychology, & Neuroscience<br>Al-Chalabi, Ammar; King's College London, Institute of Psychiatry, Psychology, & Neuroscience<br>Chaudhuri, Ray; King's College London, Institute of Psychiatry, Psychology & Neuroscience; King's College Hospital NHS Foundation Trust, Parkinson's Foundation Centre of Excellence<br>Weston, Jack; Novoic Limited<br>Fristed, Emil; Novoic Limited<br>Podlewska, Aleksandra; King's College London, Institute of Psychiatry, Psychology, & Neuroscience; King's College Hospital NHS Foundation Trust, Parkinson's Foundation Centre of Excellence<br>Awogbemila, Olabisi; King's College Hospital NHS Foundation Trust, Parkinson's Foundation Centre of Excellence<br>Young, Allan; King's College London, Institute of Psychiatry, Psychology, & Neuroscience

**Primary Subject Heading**:	Neurology
Secondary Subject Heading:	Mental health
Keywords:	Dementia < NEUROLOGY, Depression & mood disorders < PSYCHIATRY, Motor neurone disease < NEUROLOGY, Parkinson-s disease < NEUROLOGY

Protocol for RHAPSODY: a longitudinal observational study examining the feasibility of speech phenotyping for remote assessment of neurodegenerative and psychiatric disorders

Elliot Hampsey[*1], Marton Meszaros*[*2], Caroline Skirrow[2], Rebecca Strawbridge[1], Rosie H Taylor[1], Lazarus Chok[2], Dag Aarsland[1], Ammar Al-Chalabi[1], K Ray Chaudhuri[1, 3], Jack Weston[2], Emil Fristed[2], Aleksandra Podlewska[1, 3], Olabisi Awogbemila[3], Allan H Young[1].*

*EH & MM contributed equally to this manuscript.

[1] Institute of Psychiatry, Psychology, & Neuroscience, King's College London, London, UK

[2] Novoic Limited, London, UK

[3] Parkinson's Foundation Centre of Excellence at King's College Hospital NHS Foundation Trust, London, UK

Correspondence to: Mr Elliot Hampsey; elliot.hampsey@kcl.ac.uk

For peer review only

Abstract

Introduction: Neurodegenerative and psychiatric disorders (NPDs) confer a huge health burden, which is set to increase as populations age. New, remotely delivered diagnostic assessments that can detect early-stage NPDs by profiling speech could enable earlier intervention and fewer missed diagnoses. The feasibility of collecting speech data remotely in those with NPDs should be established.

Methods and analysis: The present study will assess the feasibility of obtaining speech data, collected remotely using a smartphone app, from individuals across three NPD cohorts; neurodegenerative cognitive diseases (n=50), other neurodegenerative diseases (n=50), and affective disorders (n=50), in addition to matched controls (n=75). Participants will complete audio-recorded speech tasks and both general and cohort-specific symptom scales. The battery of speech tasks will serve several purposes, such as measuring various elements of executive control (e.g., attention and Short-term memory), as well as measures of voice quality. Participants will then remotely self-administer speech tasks and follow-up symptom scales over a 4-week period. The primary objective is to assess the feasibility of remote collection of continuous narrative speech across a wide range of NPDs using self-administered speech tasks. Additionally, the study evaluates if acoustic and linguistic patterns can predict diagnostic group, as measured by the sensitivity, specificity, Cohen's kappa, and area under the receiver operating characteristic curve of the binary classifiers distinguishing each diagnostic group from each other. Acoustic features analysed include, mel-frequency cepstrum coefficients, formant frequencies, intensity, and loudness, whereas text-based features such as number of words, noun, and pronoun rate, and idea density will also be utilised.

Ethics and dissemination: The study received ethical approval from the HRA and Health and Care Research Wales (REC reference: 21/PR/0070). Results will be disseminated through open access publication in academic journals, relevant conferences, and other publicly accessible channels. Results will be made available to participants upon request.

Study registration number: NCT04939818.

Keywords: Dementia; Depression & Mood Disorders; Motor Neurone Disease; Parkinson's Disease

Word count: 3998

Strengths and limitations of this study

- Remote, partially self-administered speech-battery enabling use by those with difficulty accessing in-person healthcare.
- Speech battery and study design uniquely allows for investigation of the same tasks across a wide number of indications.
- Repeated assessment allows for examination of test reliability, and intra- and inter- subject variability across key clinical groups.
- Limited sample size.
- Short follow-up period limits scope for longitudinal disease monitoring.

Introduction

Healthcare costs of neurological and psychiatric disorders

Neurological and psychiatric disorders affect 20% of older people[1], at an estimated cost to the UK of >£68b annually[2-5]. With over 65's estimated to make up over a quarter of the UK population by 2050[6], the burden of NPDs on society will increase dramatically. Alzheimer's Disease accounts for 50-70% of dementia cases, with an estimated £34.7b annual healthcare cost in the UK[2]. Dementia with Lewy Bodies (DLB) accounts for 15-20% of all dementia cases[7-9], affecting >100,000 people in the UK. DLB is a much more rapidly progressing disease, with a median survival of 3.72 years post diagnosis[10].

Whilst less prevalent than dementias, Parkinson's disease (PD) and Motor Neurone Disease (MND) confer extensive costs due to high morbidity and mortality. PD affects >160,000 people in the UK[11], costing the NHS >£2.2b annually[12], and over 80% may progress to dementia in the long term while the rate is higher in those with the cholinergic subtype[13]. MND affects about 5,000 people in the UK[14] with patients suffering severe impairments in addition to considerable lifespan reduction[15].

Although affective disorders typically onset during one's late 20's[16], they are also marked by recurrence throughout the lifespan[17]. The World Health Organisation reports major depressive disorder (MDD) to have the highest disability burden of all conditions internationally[18], incurring a care cost in the UK of >£23.8b annually[4]. Although less prevalent (1-4.5% lifetime prevalence[19, 20]), the burden of bipolar disorder remains considerable, costing the NHS £1.6b annually[5]. People with bipolar disorder have an average of 10-20 years reduced lifespan compared to the general population, due in part to the 20% of people with bipolar disorder who die by suicide[21].

Diagnostic challenges

NPDs are complicated by challenges to accurate and early diagnosis. Estimates suggest that approximately 50% of depressive episodes go undetected[22], whilst >60% of MDD and >90% of bipolar disorder patients may be misdiagnosed[23]. Similarly, ≥50% of those who show evidence of DLB post mortem were not diagnosed with the disease during life[24], with one study finding that 39.5% of those diagnosed clinically as free from Alzheimer's disease met the minimum histopathological threshold[25]. High rates of diagnostic errors in bipolar disorder lead to the delaying of a correct diagnosis by 5.7 years[26], with many waiting for more than a decade[27]. Ostensibly, mis/delayed diagnosis can impair treatment provision, exacerbate course of illness, limit treatment options, and reduce quality of life[28].

Overlap between the clinical presentation of NPDs makes diagnosis challenging. For example, cognitive impairment, the hallmark symptom of prodromal AD, is also seen in all DLB patients[29] as well as in >80% of PD patients across all motor stages of the condition[30] and is common in people with depression. The cognitive assessments used to screen for dementia are inadequate, leaving 32% of early-stage AD[31] and 50% of DLB patients[32] undiagnosed. Diagnosing other neurodegenerative disorders such as PD is particularly challenging, as there are no definitive tests[33]. Physicians instead rely on often error-prone

For peer review only - http://bmjopen.bmj.com/site/about/guidelines.xhtml

clinical judgement[34], with an estimated 20% of those with PD who have come to medical attention going undiagnosed[35].

Even when diagnoses are made correctly, this can be years after symptoms have begun. Failure to intervene early is associated with a more severe impact on quality of life, such as memory, motor, and psychiatric disturbances[36-39].

Digital and remote assessment strategies

Uptake of digital and remote assessments methods has accelerated during the SARS-CoV-2 global pandemic, both in research and clinical practice[40,41]. Digital health technologies hold promise for reducing burden and improving access for those whom travel to medical centres would be laborious, stressful, or financially challenging[42].

Digital technology can help to enhance certain aspects of assessment practices. Higher frequency assessment, with automated administration and completed remotely, can allow for more detailed assessment of behaviour and cognition over time. Higher frequency remote assessments have been successfully deployed in mood disorders and other psychiatric conditions[43,44], and in neurological conditions, including MCI, mild AD[45] , and PD[46,47]. These studies also typically report moderate to high levels of adherence to remote assessment and good acceptability of this method of assessment. Furthermore, digital speech capture can help to enrich analyses with more advanced text similarity analyses[48] and automated extraction of language features commonly evaluated during connected speech[49], and furthermore incorporate vocal and acoustic features, which are sensitive to clinical status[50-53].

Whilst holding promise for improving convenience and access, there is concern regarding whether digital assessment is particularly challenging for certain populations, for example, in those with dementia or cognitive impairment[54]. Data integrity may be compromised by participants misunderstanding instructions (which are not or cannot be prompted for correction), or due to environmental/contextual limitations, such as distractions or ambient noise[47]. Additional care is required in the analysis of high-frequency assessments, which needs to take into account the autocorrelation of repeated observations within individuals, and potential variation within individuals over time[47].

Novel strategies to improve illness recognition

Artificial intelligence-based techniques have shown efficacy in analysing medical data in diagnosis and could potentially detect and translate subtle, early changes in speech into predictive diagnostic models[55]. This would assist care providers and their patients, who are likely to benefit from objective profiling biomarkers that are capable of disambiguating clinically similar NPDs. Speech/language alterations in NPDs are promising universal biomarkers as they reflect subtle cognitive, motor and mood changes[56-59]. As speech is among the first modalities to be affected in NPDs[56,57,60-62], developing speech/language-based profiling biomarkers will also aid in early diagnosis of NPDs. Herewith, we describe the research protocol of the “Rhapsody” study, funded by the National Institute for Health Research, which aims to assess the feasibility of recording and detecting changes in patterns of speech across a range of high burden neurological and psychiatric disorders (NPDs).

Study objectives

The primary objective of the study is to evaluate the feasibility of eliciting continuous narrative speech to collect speech data remotely in three groups of NPDs: neurodegenerative cognitive disorders, other neurodegenerative disorders (PD and MND), and affective disorders. We hypothesise that due to the simple speech-based interface, participants will be able to engage with and provide speech data during virtual study visits and remote assessments. Basic feasibility will be assessed via the average length of speech elicitation for each speech task (in seconds) during the first week of self-assessment. Additionally,

For peer review only - http://bmjopen.bmj.com/site/about/guidelines.xhtml

we will examine the proportion of participants in whom narrative speech lasting at least 20 seconds is elicited within each group.

The study also has secondary objectives concerning the feasibility of using speech tasks to collect speech data in the remote setting, such as evaluating reliability of repeated assessments across related (comparing virtual visits vs. fully remote assessments) or repeated tasks (parallel variants administered across days), examining intra- and inter-individual variance. We will also evaluate adherence to daily remote assessments, and participant-rated usability of the remotely administered application, measured via a brief usability questionnaire completed on the app.

Further, the study will examine whether acoustic and linguistic patterns can be used to distinguish from group-specific control participants, and from other clinical indications, and whether this is impacted by relevant contextual or disease information covariates.

Methods and analysis

Design

The Rhapsody study is an observational longitudinal study during which participants remotely complete a set of tasks designed to elicit speech at baseline and during the 4-week follow-up period. Participant-rated questionnaires of clinical symptoms are completed at baseline and repeated at follow-up weeks 2 and 4.

Participants

Potential participants will be identified through local clinical services, cohort-specific internal databases, advertisements on a variety of social media platforms, and dedicated websites for research participation. Participant recruitment began on the 15th July 2021 and will continue to 30th June 2022.

Participants will meet group-specific eligibility criteria for neurodegenerative cognitive disorders (n=50), or other neurodegenerative motor disorders (n=50), or affective disorders (n=50), in addition to matched controls (n=75). The sample size was based on a review of feasibility study group sizes[63]. Individuals must be native English speakers, have access to a smartphone and personal computer/laptop which can connect to the internet and is capable of audio recording as well as running an operating system of macOS X with macOS 10.9 or later; or Windows 7 or above. Exclusion criteria include: current substance use disorder, stroke within the last 2 years, transient ischemic attack or unexplained loss of consciousness within the last 12 months, or current risk of suicide. The additional group-specific eligibility criteria are:

Group 1: Neurodegenerative cognitive disorders (n=50): Participants must be aged 50-85, with a score of 20-30 on The Telephone Interview for Cognitive Status – modified[64] (TICS-M) and one of the following clinical diagnoses (within the last 5 years):

A. MCI due to Alzheimer's Disease or mild Alzheimer's dementia as per the National Institute of Aging - Alzheimer's Association core clinical criteria (2011)[65].
B. Probable or possible Dementia with Lewy Bodies as per The Dementia with Lewy Bodies Consortium definition[66].
C. Cognitive Impairment (not due to Alzheimer's/DLB), either diagnosed with behavioural variant fronto temporal dementia[67], semantic variant primary progressive aphasia or non-fluent variant primary progressive aphasia[68], vascular dementia[69] , or unspecified MCI.

Group 2: Other neurodegenerative disorders (n=50):

A. Participants with Motor Neuron Disease must be aged 18-85 and score Stage 3 or less on the King's ALS Staging system[70].

For peer review only - http://bmjopen.bmj.com/site/about/guidelines.xhtml

B. Parkinson's disease participants must be within 5 years of a diagnosis of idiopathic Parkinson's Disease according to the UK Brain Bank Criteria, aged between 30 and 85, and have a Hoehn and Yahr stage[71] of ≤2.

Group 3: Affective disorders (n=50): Participants with affective disorders must be aged 18-85, diagnosed with either major depression or bipolar disorder, in a current depressive episode according to DSM-IV criteria as assessed by the Mini international Neuropsychiatric Interview (M.I.N.I) v5.0[72], of at least moderate severity as assessed on the Clinical Global Impressions Scale[73].

Group 4: Unaffected Controls (n=75): Approximately 25 unaffected control participants will be recruited for each cohort, matched for gender, age and education levels. Participants will be in otherwise good health; they may experience mild disorders (of metabolic, respiratory, immunological, cardiologic, and metabolic origin) that do not impair daily functioning.

<u>Data collection procedures</u>

Study procedures are summarised in figure 1. Potential participants will be provided with a patient information sheet by the research team, to be followed by the study visit ≥24 hours later. Participants providing consent will complete the study visit via video conferencing software. Participants will first complete the screening and baseline assessment phases, which are completed in a single sitting.

During the screening phase, relevant demographic information, medical history, and information on current medications are obtained, as well as ruling out visual or hearing difficulties that would serve as exclusion criteria. Some cohort-specific assessments (see table 1) will also be used to determine eligibility. Participant eligibility is then evaluated against inclusion/exclusion criteria, with ineligible individuals exiting the study at this stage. Those who are eligible to continue will then complete the baseline data collection phase during the same teleconference.

[Figure 1 here]

Study procedure	Screening/ baseline	Self-assessments			
		Week 1	Week 2	Week 3	Week 4
Medical History, Concomitant Medication Overview and Demographics	X				
Telephone Interview for Cognitive Status – modified (Group 1 only)	X				
The Mini-International Neuropsychiatric Interview [a] (psychiatric diagnostic evaluation) (Group 3 only)	X				
Clinical Global Impression (Group 3 only)	X				
Speech/language assessments (see table 2)	X	X	X	X	X
Inventory of Depressive Symptomatology – Clinician Rated (Group 3 only)	X				
Patient Health Questionnaire – 9 item*	X		X		X

For peer review only - http://bmjopen.bmj.com/site/about/guidelines.xhtml

Generalised Anxiety Disorder Assessment – 7 item*	X		X		X
Maudsley 3-Item Depression Visual Analogue Scale*	X		X		X
Altman Self-Rating Mania Scale* (Groups 2 and 3 only)	X		X		X

Table 1. Screening tools and questionnaires completed at baseline and follow-up
*Patient-rated
[a]Suicide questionnaire administered if participant scores ≥10 on the Patient Health Questionnaire – 9 item

A. [All groups]: The Patient Health Questionnaire – 9-item[74] is a short, self-report tool which uses a 4-point Likert scales to assess 9 items relating to depressive symptomology.
B. [Group 3]: The Mini-International Neuropsychiatric Interview (M.I.N.I) is a structured interview for mental health diagnoses according to the Diagnostic and Statistical Manual of Mental Disorders criteria. The suicidality scale will be completed by any participant scoring ≥10 on the Patient Health Questionnaire – 9-item.
C. [Group 3]: The Clinical Global Impressions Scale is a 3-item, researcher-administered tool that provides an overall 'global' judgement concerning the participant's severity of depressive illness.
D. [Group 1]: The Telephone Interview for Cognitive Status – modified is a short, researcher-administered assessment of cognitive functioning validated for use over the phone.
E. [Group 3]: The Inventory of Depressive Symptomatology (30-item Clinician Rated)[75] is a researcher-rated instrument that provides detailed assessment of depression severity over the previous 7 days.

<u>Verbal cognitive and speech tasks</u>
All participants will complete the same sequence of verbal cognitive and speech assessments. An overview of speech tasks is provided in table 2. Some of the tasks are optimised to elicit continuous narrative speech, such as reading text and recalling a presented story. Others elicit specific categories of words, or repetition of sentences or sounds.

These tasks combined allow for evaluation of a range of cognitive, linguistic, and acoustic features elicited during spoken responses. Differential impairments are expected across groups and tasks, for example, whilst neurodegenerative cognitive disorders such as AD are commonly associated with anomia and decreased information content, other neurodegenerative conditions such as PD show greater impairment in volume and articulation[53,56]. Language-based changes have also been noted in PD including, rates of pauses, phrase length, and changes in sentence generation and construction[76]. Similarly, cognitive and speech changes in mood disorders are commonly described[52,77-79].

The consistent use of tasks across groups allows for a head-to-head comparison of task performance and speech measures for different clinical indications and different tasks. The tasks are selected due to a rich research background showing their involvement in specific clinical indications (described in table 2). However, with a few notable exceptions, cross-diagnostic evaluations, are not commonly reported.

**Speech and cognitive tasks**	**Task details**	**Task characteristics and selected evidence from prior research.**
Count 1-10 or 10-1	Participants are recorded counting from 1 to 10 or from 10 to 1.	The counting task was used as a brief familiarisation task for participants.

For peer review only

The Automatic Story Recall Task (ASRT) with immediate and delayed recall	The ASRT has 36 parallel story variants (18 'short' and 18 'long' stories), presented at a steady reading rate and matched for linguistic and discourse measures, including number of words, number of sentences, number of dependent clauses, mean sentence length, and ratio of dependent clauses to t-units[45]. Pre-recorded stories are played to the participants, who are be asked to recall the stories in as much detail as possible immediately and again after a delay.	Story recall is often used to evaluate verbal episodic memory. Prior meta analytic evidence suggests that lesser impairment would be expected in story recall performance for individuals with DLB than those with AD-MCI[80]. A smaller head-to-head research study indicates that lesser impairment may be expected for individuals with PD than AD-MCI[81]. Meta analytic evidence suggests that the task may be sensitive to mood disorders, with individuals with a history of depression having task performance deficits in story recall, with large effect sizes reported[82].
Category Fluency Task	Participants will be given a category and required to give as many examples of that category as they can in 60 seconds. Participants will complete parallel variants of this task, commonly implemented in the research literature, including animals, vegetables, fruits.	Category fluency evaluates semantic memory function and taps into attentional and executive functions. Impairments in category fluency have been noted in a rage of the clinical indications, including MCI/AD and DLB[83], PD[82], MND[84] MDD[82] and BD[85], but with different reported effect sizes.
Letter Fluency Task	Participants will be given a letter of the alphabet and required to give as many examples of words beginning with that letter as they can in 60 seconds. Letters prompted include F, A and S.	Letter fluency tasks evaluate lexical access through the phonological route and are believed to more strongly depend on executive functions than category fluency measures. Prior research shows that task performance is impaired in adults with MCI[86], PD[83] MND[84], following MDD[82] and in BD[85], but with different reported effect sizes.
Action Fluency Task	Participants are tasked with naming as many of examples of things that "people do", i.e., generate verbs such as "run" or "work", in 60 seconds.	Impairments in verb fluency tasks are thought to more greatly reflect fronto-striatal neuropathology and neurochemical deterioration known to occur with progression of PD[87]. A systematic review[88] reports more prominent impairment for action fluency than for category fluency for PD, differential task performance for AD and DLB participants in action fluency, and no difference in action fluency for comparisons of MCI and healthy control participants.
Digit span forward and backwards	In this abbreviated digit span task, in forwards digit span, a series of 5 digits is presented to participants (e.g.: 8-3-1-9-6), which they are asked to repeat back in the	Digit span tests are associated with auditory attention and short-term memory function, with greater reliance on working memory for the backwards span variant.

For peer review only - http://bmjopen.bmj.com/site/about/guidelines.xhtml

	same order. In backwards digit span a series of 5 digits is presented to participants (e.g.: 8-3-1-9-6), which they are asked to repeat in backwards order (e.g.: 6-9-1-3-8). Forwards and backwards digit span were completed three times each with three different 5-digit sequences.	Meta-analyses show greater impairments in backwards span than in forwards span for MCI/AD[80]; PD[89]. In affective disorders, meta analyses have emphasised deficits in backwards span in the absence of deficits in forward digit span[77,82].
Stroop	In this abbreviated Stroop task, participants are first presented with a grid of 50 colours written out in text (e.g., "BLUE") and asked to read these back as quickly as they can. They are then presented with a panel of colours presented in blocks and are asked to name these as quickly as possible. Finally, they are presented with a panel of 50 words typed with the typeface in a colour incongruent with the written word (e.g. "GREEN" written in the colour red). They are asked to state the colour of the letters as quickly as possible.	The Stroop test is an extensively used test to evaluate inhibition to verbal interference. Prior meta analyses have shown impairments in Stroop interference performance in individuals with amnestic MCI[90]; PD[83]; MND[84]; MDD[77] and BD[78].
Procedural Discourse Questions	Participants are tasked with describing, in as much detail as possible, how they would go about doing the dishes by hand (in main assessment), how they would go about posting a letter, and how they would make a cup of tea (each assessed on one occasion during remote assessments).	The procedural discourse task elicits naturalistic speech, used to express temporal and hierarchical steps in a behavioural sequence. The task has been found to be sensitive to speech differences in individuals with MCI and mild AD in comparison with healthy control participants[91].
Picture Description Task	Participants verbally describe a picture in as much detail as they can. Four different pictures will be used, the first being the 'cookie theft' picture taken from the Boston Diagnostic Aphasia Examination battery[92] administered during main assessment), and the other two being the 'rescue' picture[93] and two simple emotion eliciting pictures administered during remote assessments[94].	The picture description task produces a structured output that can be scored according to the completeness of response, and is useful for measuring and detecting differences in language content, syntax, pragmatics and acoustic features[49]. Research shows changes in this task for individuals with MCI and AD dementia, lesser impairment in individuals with depression, and differences in speech error corrections between AD and PD groups (reviewed by Mueller and colleagues[49]).
Sequence Narration task	Participants must describe their narrative interpretation of a series of images presented as a storyboard, taken from the 'Argument' sequence[93]. Participants have one minute to look at the picture sequence and are then asked to tell the story represented by the picture sequence.	The task produces a relatively structured output, where analysis outputs can include completeness of the narrative. The task is also used to evaluate lexico-semantic deficits, and syntactic complexity. The pictorially provided structure is thought to

For peer review only - http://bmjopen.bmj.com/site/about/guidelines.xhtml

		place a decreased load on working memory[95].
Reading a Script	Participants will read a short passage aloud: "The boy who cried wolf"[96]. A Passage designed for phonetic description and acoustic research on varieties of English.	This task allows the evaluation of pronunciation variations in the English language in different accents and dialects.
Sustained Phonation Task	Participants are required to phonate several sounds (/a:/, /i:/ and /u:/) for as long and steadily as possible, in one breath.	Sustained phonation allows for the measurement of voice quality. Changes in vowel articulation has been noted as a potential early marker for PD, and research shows changes in the audio speech measures distinguish PD participants from controls and a likely increase in voice impairment following developmental trajectory of the individuals.
Sentence reading task	During sentence repetition, a sentence (containing 11 or 15 words) is presented auditorily to the participant is asked to must repeat it back in the same way and in one breath. The sentences each contain two /u:/, two /i:/ and two /a:/ long corner vowels to measure vowel space area, following voiceless consonants. During each assessment, two different sentences are presented	Sentence repetition allows for the measurement of voice quality within a more naturalistic speech response. Research indicates that sentence repetition may be more sensitive to audio speech changes in PD than sustained phonation[97].
Syllable Repetition Task	Participants are required to repeatedly phonate a syllable as quickly as possible for one breath. They are first asked to compete this for the syllable /ba/ and then for three consecutive syllables /pa/ /ta/ /ka/.	These tasks assess difficulties with phonology, articulation and working memory. Prior research shows changes in acoustic features in the /pa/ /ta/ /ka/ task in PD (including jitter, pause rates, intensity variation, alternating motion rate and pitch variation[97]. Other research shows changes acoustic features (including alternating motion rate, jitter, frequency and an overall variable) for MND in repeated phonation of the syllable /ba/[98].

Table 2. Overview of speech tasks used in RHAPSODY (main and/or remote assessments)

Speech tasks will be administered and recorded using the application, with all participants completing the same set of speech tasks. Participants will be supported with using the Novoic application on their smartphone. Audio recordings will be transferred to a secure server and then deleted from the smartphone device.

Recordings of the combined screening and baseline visit will also be made simultaneously by video conferencing software. Zoom (https://zoom.us) will be used due to its ability to turn off audio manipulation effects. The main assessment phase begins with a series of speech tasks, self-recorded using

For peer review only - http://bmjopen.bmj.com/site/about/guidelines.xhtml

a smartphone application, that the researcher guides the participant to complete. An overview of the specific tasks completed in the main assessment is provided in table 3.

Following the completion of the main sequence speech tasks, all participants will then be asked to complete online self-rated questionnaires via the Qualtrics platform (www.qualtrics.com)(see table 1):

A. The General Anxiety Disorder Assessment 7-item[99] is a brief tool for assessing symptoms of anxiety. Participants rate 7 items relating to core symptoms of anxiety on a 4-point Likert scale concerning the frequency at which those symptoms occur.

B. The Maudsley 3-Item Depression Visual Analogue Scale[100] is a researcher-rated assessment of depressive illness which detects symptom severity and suicidality.

C. The Altman Self Rating Mania Scale[101] is a 5-item instrument designed to self-assess the presence and severity of manic and hypomanic symptoms by comparing how they feel currently to their non-affected baseline state.

Participants will then complete follow up data collection over a period of 4 weeks using the smartphone application. Participants will be notified via the mobile application to complete 12 brief (approximately 15 minute) unsupervised speech and cognition assessments over the month following the baseline visit. Participants will receive a different set of speech tasks each day from the application's built-in task-bank, including the same main speech and cognition tasks described above, and abbreviated test of executive function (Stroop, digit span).

Additional, brief self-report measures, incorporated into remote assessments, included:

- A 4-item questionnaire on the usability of the smartphone application
- A brief, 3-minute-long questionnaire on whether the participant has any visual or hearing impairments, and whether they had related difficulties with the administered tasks.
- A baseline 4-item questionnaire: examining typical mood, sleep, mind wandering and effort
- A brief, daily 4-item questionnaire on current state: mood, sleep, mind wandering and effort

The remote assessment schedule is provided in table 3. Participants will also complete follow-up questionnaires online via Qualtrics during weeks 2 and 4, which are shown in table 1.

	**Main (supervised) assessment**	**Remote assessment day**											
**Task**		**1**	**2**	**3**	**4**	**7**	**8**	**9**	**10**	**11**	**14**	**21**	**28**
Counting 1-10 or 10-1	X	X	X	X	X	X	X	X	X	X	X	X	X
Automated Story Recall Task immediate and delayed recall	X	X	X	X	X	X	X	X	X	X	X[R]	X[R]	X[R]
Category fluency	X	X					X				X[R]	X[R]	X[R]
Letter fluency	X		X					X			X[R]	X[R]	X[R]
Action fluency	X										X[R]		

For peer review only - http://bmjopen.bmj.com/site/about/guidelines.xhtml

Digit span forward and backward				X					X				
Stroop						X							
Procedural discourse	X				X					X			
Picture description	X		X					X					
Sustained phonation	X										X[R]		
Syllable repetition	X	X[R]									X[R]		
Sentence repetition	X	X[R]									X[R]		
Sequence narration	X												
Reading a script	X												X [R]
Usability questionnaire	X		X							X			
Vision and hearing questionnaire	X			X									
Baseline mood, sleep, attention & effort questionnaire	X	X											
Daily mood, sleep, attention & effort questionnaire	X	X	X	X	X	X	X	X	X	X	X	X	X

Table 3. Assessments completed during main (supervised) assessment, and remotely via the application on the participants' own smartphones
Remote assessment day corresponds to the number of days after the virtual visit. Test order is shown in descending order. All Automated Story Recall Task immediate and delayed recall assessments are completed with brief distractor tasks occurring in-between. [R] Repeated stimuli

For peer review only

Statistical analysis plan

The main analysis objectives of the study include examining: (1) feasibility of eliciting and collecting speech data within different clinical groups, (2) reliability, and intra and inter-subject variance of speech task performance, (3) adherence to remote unsupervised assessments, (4) app usability, and (5) to evaluate whether acoustic and linguistic patterns can be used to distinguish between clinical indications and controls.

Feasibility will be assessed as the length of speech generated during speech elicitation tasks. The primary endpoint is calculated as the number of participants who successfully complete Automated Story Recall Task assessments as a fraction of the disease cohort, which may indicate likely suitability of this procedure within each disease group. Participants are considered successful where at least one of the

For peer review only - http://bmjopen.bmj.com/site/about/guidelines.xhtml

immediate story recalls produces a spoken response spanning ≥20 seconds from the first to the last word. The effect of demographic confounders (age, sex, and education) on task feasibility will be evaluated.

Reliability will be measured by examining the stability of equivalent tasks completed over the assessment period. This will include examining reliability across parallel test variants that vary by assessment day and by setting (baseline vs. remote follow-up).

Practice and learning effects of parallel test variants over repeated assessment will be characterised with linear mixed effects models. Correlational analysis will be completed between parallel test variants, and intraclass correlations will be carried out to examine test-retest reliability of the same test variants administered across days and between settings (virtual study visit vs. remote assessment). Coefficients of individual agreement will be used as a measure of inter-participant and intra-participant variability.

Usability will be examined via the scores on the usability questionnaire for each disease cohort and their matched controls, to examine disease cohort-specific problems or difficulties in completing remote assessments. Adherence will be defined as the proportion of participants engaging in daily remote assessments in each indication. Data will be analysed using t-tests, ANOVAs or non-parametric equivalents, as appropriate. Age and education differences between groups will be controlled for as appropriate.

For the endpoints which require training classifiers or regressors to make predictions, the entire data set will be used for both training machine learning models and validating their statistical properties. Machine learning methods used may include supervised, unsupervised, semi-supervised and reinforcement learning methods for forming intermediate representations of the data and performing the final classification/regression analyses. Speech measures will be derived using several methods from the fields of signal/speech processing and natural language processing to investigate which speech markers are most predictive of clinical status. Some of the planned methods for analyses are described here, but in this broad rapidly evolving field, others may be used where suitable and as they become available.

The sample rate, bit rate, and codec compression of smartphones along with speech intelligibility measures will be used to probe dependence of speech features on audio quality measures. Data from the mPower study suggests that phonation data from a bring-your-own-device setup is sufficiently consistent to predict motor disorders to some degree (see e.g., Lenain et al., 2020[102]).

Acoustic measures will be extracted via Surfboard, an automated audio feature extraction library[102], which extracts a range of acoustic speech features (such as mel-frequency cepstrum coefficients, F0 contour, formant frequencies, intensity, loudness); and VQP, a self-supervised contrastive model for non-timbral prosody[103]. Linguistic and pause analyses will be completed after automated transcription for onward textual analysis. Text features (including, number of words, noun, and pronoun rate, idea density) will be extracted using Stanza[104]. Speaking time, articulation rate, and pause information will be extracted directly from the number of words and timestamps in the transcriptions. Group prediction from text and audio features will be evaluated within the python machine learning package scikit learn[105] with k-fold cross-validation. For ASRTs, textual analyses will be completed with ParaBLEU, a paraphrase evaluation model[106], and G-match, evaluating the cosine similarity of textual embeddings between two texts[45].

Deep learning methods will be utilised, where the speech measures are derived as internal representations of the models. Methods used here will include unsupervised context-aware word embeddings, a way of distinguishing between identical words with different contextual meanings[106], and audio representation learning[103].

For peer review only - http://bmjopen.bmj.com/site/about/guidelines.xhtml

Training and validation of the ML models will be performed using cross-validation techniques. Performance of classification analysis will be characterised by the area under the receiver operating characteristic (ROC) curve (AUC), sensitivity, specificity, and Cohen's kappa. Demonstration that a binary classifier performs better than the random baseline will be done via mapping AUC estimates to a p-value via the Mann-Whitney U statistic[107]. Results will be contrasted with demographic comparisons (comprising sex, education and age) as in prior analyses[48], to evaluate the contribution of demographic imbalances.

Methodological issues and limitations

The primary potential limitation of the study is poor compliance with the self-administered speech tasks over the follow-up period. Efforts to address this potential issue include reminder notifications from the smartphone application and allowing participants to complete these self-assessments at flexible timepoints. It should also be noted that adherence to the self-administered tasks is itself an outcome measure of the study, and so missing data in this regard is informative.

Practice effects on repeated tasks are likely to have an impact on task performance. Parallel test variants, such as those for the ASRT, can minimise practice effects on repeated exposure. However, even with parallel test variants, repeated administration is likely to see task improvements over time related to repeated exposure to the same task and greater familiarity with the test structure and method[45]. For some conditions and some tasks such as category fluency, the practice effects themselves (or lack thereof) may be of interest for identifying specific diagnostic groups, such as individuals with MCI on tasks such as category fluency[108,109].

Other potential limitations include the fact that recruitment is limited by the requirement for participants to be able to access and use electronic devices for speech tasks and follow-up questionnaires, as not all patients own or have access to connected digital platforms[40], even with assistance from others.

Data management and oversight

All aspects of the study will be overseen by the Study Management Group. Primary investigators will oversee and manage all aspects of their study in their respective cohorts. Their responsibility includes monitoring adverse events in their respective cohorts and managing participant discontinuation.

Audio data will be recorded by the app and by the video conference software. Upon completion of each assessment, the audio will be securely transferred to Novoic's cloud servers, which are fully secure and compliant under relevant security standards (including ISO27001) and privacy regulations (including GDPR). Other clinical data will be stored in a secure password-protected server, compliant with all applicable laws and regulations, including ICH E6 Good Clinical Practice (GCP), EU Annex 11, GDPR.

Data access

The speech data obtained during the study will not be made available to a repository due to the potential for participant identification.

Patient and public involvement

We have involved patients and members of the public in planning of this study, having sought service users' perspectives on protocol documentation which resulted in amendments to language used and patient facing documentation. We have also implemented changes based on carers' feedback from a focus group of 10-15 individuals. We also actively collect usability feedback from service users. During dissemination, we will invite service users and carers to contribute to a public perspective on the interpretation of trial findings. Results will be made available to participants upon request.

Ethics and dissemination

For peer review only - http://bmjopen.bmj.com/site/about/guidelines.xhtml

The study received REC approval from the London - Queen Square Research Ethics Committee on the 15th April 2021, and HRA approval from HRA and Health and Care Research Wales (HCRW) on the 23rd April 2021. (REC reference: 21/PR/0070). Findings will be published in peer-reviewed journals and presented at conferences focussed on neuroscience & psychiatry, and machine learning & natural language processing. Novoic will create a project webpage containing key findings, published papers, and open-source software packages. Results will be made available to participants upon request.

The study is registered with Clinicaltrials.gov (NCT04939818).

Contributors

Mr Elliot Hampsey (ORCID ID: 0000-0001-6985-5646): contributed to the writing of the study protocol, in addition to the writing and editing of the manuscript for journal submission.
Dr Marton Meszaros (ORCID ID: 0000-0003-4937-3062): main contributor on designing the study and protocol. Contributed to writing the full study protocol and editing the manuscript for journal submission.
Dr Caroline Skirrow (ORCID ID: 0000-0001-8692-7787): contributed to the study design, writing, and editing of the manuscript for journal submission.
Dr Rebecca Strawbridge (ORCID ID: 0000-0002-2984-1124): contributed to the editing of the study protocol and manuscript for journal submission, in addition to study set up activities.
Miss Rosie Hannah Taylor (ORCID ID: 0000-0001-6742-4842): Contributed to editing of the manuscript for journal submission.
Mr Lazarus Chok (ORCID ID: 0000-0002-9514-364X): contributed to the writing of the study protocol, in addition to the editing of the manuscript for journal submission.
Professor Dag Aarsland (ORCID ID: 0000-0001-6314-216X): Contributed to study and protocol design. Principal investigator for neurodegenerative disease cohort.
Professor Ammar Al-Chalabi (ORCID ID: 0000-0002-4924-7712): Contributed to study and protocol design. Principal investigator for motor disorders cohort.
Professor K Ray Chaudhuri (ORCID ID: 0000-0003-2815-0505): Contributed to study and protocol design. Principal investigator for motor disorders cohort.
Dr Jack Weston (ORCID ID: 0000-0001-5344-7840): contributed to the writing of the study protocol, in addition to editing of the manuscript for journal submission.
Mr Emil Fristed (ORCID ID: 0000-0002-9590-7275): main contributor on designing the study and protocol. Contributed to writing the full study protocol and editing the manuscript for journal submission.
Ms Aleksandra Podlewska (ORCID ID: 0000-0002-0805-3356): Contributed to editing of the manuscript for journal submission.
Ms Olabisi Awogbemila (ORCID ID: 0000- 0002- 6814- 3097): Contributed to editing of the manuscript for journal submission.
Professor Allan H. Young (ORCID ID: 0000-0003-2291-6952): Chief Investigator. Contributed to study and protocol design and edited the manuscript for journal submission.

Acknowledgments

We would like to thank the NIHR Maudsley Biomedical Research Centre's FAST-R service and Service User Advisory Group. This report is independent research funded by the National Institute for Health Research (Artificial Intelligence, Project Rhapsody: Investigating the clinical feasibility of using AI-based deep audio and language processing techniques to diagnose neurological and psychiatric diseases, AI_AWARD01984) and NHSX. The views expressed in this publication are those of the author(s) and not necessarily those of the National Institute for Health Research, NHSX or the Department of Health and Social Care.

Funding

For peer review only - http://bmjopen.bmj.com/site/about/guidelines.xhtml

This work is supported by the National Institute of Health Research. The study has been awarded by the NHS Artificial Intelligence in Health and Care Award (grant number AI_AWARD01984).

Competing interests

R.S. has received an honorarium for speaking from Lundbeck. In the past 3 years: A.H.Y. has received honoraria for speaking from AstraZeneca, Lundbeck, Eli Lilly and Sunovion; honoraria for consulting from Allergan, Livanova and Lundbeck, Sunovion and Janssen; and research grant support from Janssen. EH declares no conflicts of interest. EF is CEO of Novoic. MM, JW and CS are employees of Novoic, and EF, MM and JW are shareholders in the company.

References

1. Mental health of older adults [Internet]. Who.int. 2021 [cited 30 September 2021]. Available from: https://www.who.int/news-room/fact-sheets/detail/mental-health-of-older-adults

2. Wittenberg R, Hu B, Barraza-Araiza L, Rehill A. Projections of older people with dementia and costs of dementia care in the United Kingdom, 2019–2040. Care Policy and Evaluation Centre Working Paper 5. 2019.

3. Weir S, Samnaliev M, Kuo T, Tierney T, Walleser Autiero S, Taylor R et al. Short- and long-term cost and utilization of health care resources in Parkinson's disease in the UK. Movement Disorders. 2018;33(6):974-981.

4. No Health Without Mental Health: a cross-government mental health outcomes strategy for people of all ages. 2011.

5. McCrone P, Dhanasiri S, Patel A, Knapp M, Lawton-Smith S. Paying the price: The cost of mental health care in England to 2026. 2008.

6. Overview of the UK population - Office for National Statistics [Internet]. Ons.gov.uk. 2021 [cited 30 September 2021]. Available from: https://www.ons.gov.uk/peoplepopulationandcommunity/populationandmigration/populationestimates/articles/overviewoftheukpopulation/august2019

7. McKeith I, Mintzer J, Aarsland D, Burn D, Chiu H, Cohen-Mansfield J et al. Dementia with Lewy bodies. The Lancet Neurology. 2004;3(1):19-28.

8. A Guide to Lewy Body Dementia. The Lewy Body Society, 2019.

9. O'Brien, J., Taylor, J., Thomas, A., Bamford, C., Vale, L., Hill, S., Allan, L., et al. Improving the Diagnosis and Management of Neurodegenerative Dementia of Lewy Body Type in the NHS (DIAMOND-Lewy Programme). NIHR Journals Library Publications https://doi.org/10.17863/CAM.65933

10. McKeith I, Rowan E, Askew K, Naidu A, Allan L, Barnett N et al. More Severe Functional Impairment in Dementia With Lewy Bodies Than Alzheimer Disease Is Related to Extrapyramidal Motor Dysfunction. The American Journal of Geriatric Psychiatry. 2006;14(7):582-588

For peer review only - http://bmjopen.bmj.com/site/about/guidelines.xhtml

11. Gumber, A., Ramaswamy, B., Ibbotson, R., Ismail, M., Thongchundee, O., Harrop, D., Allmark, P., & Rauf, A. (2017). Economic, Social and Financial Cost of Parkinson’s on Individuals, Carers and their Families in the UK. Project Report. Centre for Health and Social Care Research, Sheffield Hallam University.

12. McCrone P, Allcock L, Burn D. Predicting the cost of Parkinson's disease. Movement Disorders. 2007;22(6):804-812.

13. Marras C, Chaudhuri KR. Nonmotor features of Parkinson's disease subtypes. Mov Disord. 2016 Aug;31(8):1095-102. doi: 10.1002/mds.26510. Epub 2016 Feb 10. PMID: 26861861

14. Gowland A, Opie-Martin S, Scott K, Jones A, Mehta P, Batts C et al. Predicting the future of ALS: the impact of demographic change and potential new treatments on the prevalence of ALS in the United Kingdom, 2020–2116. Amyotrophic Lateral Sclerosis and Frontotemporal Degeneration. 2019;20(3-4):264-274.

15. Westeneng H, Debray T, Visser A, van Eijk R, Rooney J, Calvo A et al. Prognosis for patients with amyotrophic lateral sclerosis: development and validation of a personalised prediction model. The Lancet Neurology. 2018;17(5):423-433.

16. Solmi M, Radua J, Olivola M, Croce E, Soardo L, Salazar de Pablo G et al. Age at onset of mental disorders worldwide: large-scale meta-analysis of 192 epidemiological studies. Molecular Psychiatry. 2021.

17. Kessing L. Recurrence in affective disorder. British Journal of Psychiatry. 1998;172(1):29-34.

18. James S, Abate D, Abate K, Abay S, Abbafati C, Abbasi N et al. Global, regional, and national incidence, prevalence, and years lived with disability for 354 diseases and injuries for 195 countries and territories, 1990–2017: a systematic analysis for the Global Burden of Disease Study 2017. The Lancet. 2018;392(10159):1789-1858.

19. Fajutrao L, Locklear J, Priaulx J, Heyes A. A systematic review of the evidence of the burden of bipolar disorder in Europe. Clinical Practice and Epidemiology in Mental Health. 2009;5(1):3.

20. Hirschfeld R. Differential diagnosis of bipolar disorder and major depressive disorder. Journal of Affective Disorders. 2014;169:S12-S16.

21. Dome P, Rihmer Z, Gonda X. Suicide Risk in Bipolar Disorder: A Brief Review. Medicina. 2019;55(8):403.

22. Mitchell A, Vaze A, Rao S. Clinical diagnosis of depression in primary care: a meta-analysis. The Lancet. 2009;374(9690):609-619.

23. Vermani M, Marcus M, Katzman M. Rates of Detection of Mood and Anxiety Disorders in Primary Care. The Primary Care Companion for CNS Disorders. 2011.

24. Hohl U, Tiraboschi P, Hansen L, Thal L, Corey-Bloom J. Diagnostic Accuracy of Dementia With Lewy Bodies. Archives of Neurology. 2000;57(3):347.

25. Beach T, Monsell S, Phillips L, Kukull W. Accuracy of the Clinical Diagnosis of Alzheimer Disease at National Institute on Aging Alzheimer Disease Centers, 2005–2010. Journal of Neuropathology & Experimental Neurology. 2012;71(4):266-273.

For peer review only - http://bmjopen.bmj.com/site/about/guidelines.xhtml

26. Morselli P, Elgie R. GAMIAN-Europe*/BEAM survey I - global analysis of a patient questionnaire circulated to 3450 members of 12 European advocacy groups operating in the field of mood disorders. Bipolar Disorders. 2003;5(4):265-278.

27. Dagani J, Signorini G, Nielssen O, Bani M, Pastore A, Girolamo G et al. Meta-analysis of the Interval between the Onset and Management of Bipolar Disorder. The Canadian Journal of Psychiatry. 2016;62(4):247-258.

28. Rasmussen J, Langerman H. Alzheimer's Disease – Why We Need Early Diagnosis. Degenerative Neurological and Neuromuscular Disease. 2019; Volume 9:123-130.

29. Goldman J, Williams-Gray C, Barker R, Duda J, Galvin J. The spectrum of cognitive impairment in Lewy body diseases. Movement Disorders. 2014;29(5):608-621.

30. Aarsland, D., Andersen, K., Larsen, J.P., Lolk, A. & Kragh-Sørensen, P. (2003). Prevalence and characteristics of dementia in Parkinson Disease: An 8-year prospective study. Archives of Neurology, 60(3), pp.387-92.

31. NHS Digital (2020). Recorded Dementia Diagnoses January 2020. London: NHS England.

32. Price A, Farooq R, Yuan J, Menon V, Cardinal R, O'Brien J. Mortality in dementia with Lewy bodies compared with Alzheimer's dementia: a retrospective naturalistic cohort study. BMJ Open. 2017;7(11):e017504.

33. Parkinson's Disease: Clinical features and diagnosis. Journal of Neurology, Neurosurgery and Psychiatry, 79(4), pp.368-76.

34. Rizek, P., Kumar, N. & Jog, M.S. (2016). An update on the diagnosis and treatment of Parkinson disease. CMAJ, 188(16), pp.1157-65.

35. Schrag A, Ben-Shlomo Y, Quinn N. How valid is the clinical diagnosis of Parkinson's disease in the community? J Neurol Neurosurg Psychiatry. 2002 Nov;73(5):529-34. doi: 10.1136/jnnp.73.5.529. PMID: 12397145; PMCID: PMC1738115.

36. Sperling, R.A., Dickerson, B.C., Pihlajamaki, M., Vannini, P., LaViolette, P.S., Vitolo, O.V., Hedden, T., Becker, J.A., Rentz, D.M., Selkoe, D.J., & Johnson, K.A. (2010). Functional alterations in memory networks in early Alzheimer's disease. Neuromolecular Medicine, 12(1), pp.27-43.

37. Kulisevsky, J., & Pagonabarraga, J. (2009). Cognitive impairment in Parkinson's disease: tools for diagnosis and assessment. Movement Disorders, 24(8): pp.1103-10.

38. D'Onofrio, G., Sancarlo, D., Panza, F., Copetti, M., Cascavilla, L., Paris, F., Seripa, D., Matera, M.G., Solfrizzi, V., Pellegrini, F., & Pilotto, A. (2012). Neuropsychiatric symptoms and functional status in Alzheimer's disease and vascular dementia patients. Current Alzheimer Research, 9(6), pp.759-71.

39. NICE (2015). Motor Neurone Disease Briefing Paper. London: NICE.

For peer review only - http://bmjopen.bmj.com/site/about/guidelines.xhtml

40. De Marchi F, Contaldi E, Magistrelli L, Cantello R, Comi C, Mazzini L. Telehealth in Neurodegenerative Diseases: Opportunities and Challenges for Patients and Physicians. Brain Sciences. 2021;11(2):237.

41. Patel S, Mehrotra A, Huskamp H, Uscher-Pines L, Ganguli I, Barnett M. Trends in Outpatient Care Delivery and Telemedicine During the COVID-19 Pandemic in the US. JAMA Internal Medicine. 2021;181(3):388.

42. National Academies of Sciences, Engineering, and Medicine; Health and Medicine Division; Board on Health Sciences Policy; Forum on Drug Discovery, Development, and Translation. *Virtual Clinical Trials: Challenges and Opportunities: Proceedings of a Workshop*. Edited by Carolyn Shore et. al., National Academies Press (US), 23 July 2019. doi:10.17226/25502

43. Cormack F, McCue M, Taptiklis N, Skirrow C, Glazer E, Panagopoulos E et al. Wearable Technology for High-Frequency Cognitive and Mood Assessment in Major Depressive Disorder: Longitudinal Observational Study. JMIR Mental Health. 2019;6(11):e12814.

44. Goodday S, Atkinson L, Goodwin G, Saunders K, South M, Mackay C et al. The True Colours Remote Symptom Monitoring System: A Decade of Evolution. Journal of Medical Internet Research. 2020;22(1):e15188.

45. Skirrow C, Meszaros M, Meepegama U, Lenain R, Papp K, Weston J et al. Validation of a remote and fully automated story recall task to assess for early cognitive impairment in older adults: a longitudinal case-control observational study. 2022.

46. Broen M, Marsman V, Kuijf M, Van Oostenbrugge R, van Os J, Leentjens A. Unraveling the Relationship between Motor Symptoms, Affective States and Contextual Factors in Parkinson's Disease: A Feasibility Study of the Experience Sampling Method. PLOS ONE. 2016;11(3):e0151195.

47. Omberg L, Chaibub Neto E, Perumal T, Pratap A, Tediarjo A, Adams J et al. Remote smartphone monitoring of Parkinson's disease and individual response to therapy. Nature Biotechnology. 2021.

48. Fristed E, Skirrow C, Meszaros M, Lenain R, Meepegama U, Cappa S et al. Evaluation of a speech-based AI system for early detection of Alzheimer's disease remotely via smartphones. 2021.

49. Mueller K, Hermann B, Mecollari J, Turkstra L. Connected speech and language in mild cognitive impairment and Alzheimer's disease: A review of picture description tasks. Journal of Clinical and Experimental Neuropsychology. 2018;40(9):917-939.

50. Codina-Filbà, J., Escalera, S., Escudero, J., Antens, C., Buch-Cardona, P., Farrús, M. (2021). Mobile eHealth Platform for Home Monitoring of Bipolar Disorder. In: , *et al.* MultiMedia Modeling. MMM 2021. Lecture Notes in Computer Science(), vol 12573. Springer, Cham. https://doi.org/10.1007/978-3-030-67835-7_28

51. Martínez-Nicolás I, Llorente T, Martínez-Sánchez F, Meilán J. Ten Years of Research on Automatic Voice and Speech Analysis of People With Alzheimer's Disease and Mild Cognitive Impairment: A Systematic Review Article. Frontiers in Psychology. 2021;12.

52. Low D, Bentley K, Ghosh S. Automated assessment of psychiatric disorders using speech: A systematic review. Laryngoscope Investigative Otolaryngology. 2020;5(1):96-116.

For peer review only - http://bmjopen.bmj.com/site/about/guidelines.xhtml

53. Smith K, Caplan D. Communication impairment in Parkinson's disease: Impact of motor and cognitive symptoms on speech and language. Brain and Language. 2018;185:38-46.

54. Sano M, Lapid M, Ikeda M, Mateos R, Wang H, Reichman W. Psychogeriatrics in a world with COVID-19. International Psychogeriatrics. 2020;32(10):1101-1105.

55. Patel, U. K., Anwar, A., Saleem, S., Malik, P., Rasul, B., Patel, K., ... & Arumaithurai, K. (2021). Artificial intelligence as an emerging technology in the current care of neurological disorders. Journal of neurology, 268(5), 1623-1642.

56. Boschi, V., Catricalà, E., Consonni, M., Chesi, C., Moro, A., & Cappa, S.F. (2017). Connected Speech in Neurodegenerative Language Disorders: A Review. Frontiers in psychology, 8, 269.

57. Engelman, M., Agree, E.M., Meoni, L.A. & Klag, M.J. (2010). Propositional density and cognitive function in later life: Findings from the precursors study. J Gerontol., 65, pp.706-11.

58. Fraser, Meltzer, Ruudzicz. Linguistic features identify alzheimers disease in narrative speech. J Alzheimer's Dis. 2016;49:407–22.

59. Cummins, N., Scherer, S., Krajewski, J., Schnieder, S., Epps, J., & Quatieri, T. F. (2015). A review of depression and suicide risk assessment using speech analysis. Speech communication, 71, 10-49.

60. Riley, K., Snowdon, D.A., Desrosiers, M.F. & Markesbery, W.R. (1996). Early life linguistic ability, late life cognitive function, and neuropathology: findings from the Nun Study. Neurobiology of Aging, 26(3), pp.341-7.

61. Cuetos, F., Arango-Lasprilla, J.C., Uribe, C., Valencia, C. & Lopera, F. (2007). Linguistic changes in verbal expression: A preclinical marker of Alzheimer's Disease. J Int Neuropsychol Soc., 13, pp.433-9.

62. Snowdon, D.A., Kemper, S.J., Mortimer, J.A., Greiner, L.H., Wekstein, D.R. & Markesbery, W.R. (1996). Linguistic ability in early life and cognitive function from the nun and Alzheimer's Disease in late life study. JAMA, 275, pp.528-32.

63. Hertzog M. Considerations in determining sample size for pilot studies. Research in Nursing & Health. 2008;31(2):180-191.

64. Welsh, K. A., Breitner, J. C., & Magruder-Habib, K. M. (1993). Detection of dementia in the elderly using telephone screening of cognitive status. *Neuropsychiatry, Neuropsychology, & Behavioral Neurology, 6*(2), 103–110.

65. Albert M, DeKosky S, Dickson D, Dubois B, Feldman H, Fox N et al. The diagnosis of mild cognitive impairment due to Alzheimer's disease: Recommendations from the National Institute on Aging-Alzheimer's Association workgroups on diagnostic guidelines for Alzheimer's disease. Alzheimer's & Dementia. 2011;7(3):270-279.

66. McKeith I, Boeve B, Dickson D, Halliday G, Taylor J, Weintraub D et al. Diagnosis and management of dementia with Lewy bodies. Neurology. 2017;89(1):88-100.

For peer review only - http://bmjopen.bmj.com/site/about/guidelines.xhtml

67. Rascovsky K, Hodges JR, Knopman D, Mendez MF, Kramer JH, Neuhaus J, Miller BL, et al. Sensitivity of revised diagnostic criteria for the behavioural variant of frontotemporal dementia. *Brain.* 2011;134:2456–2477.

68. Mesulam M. Primary progressive aphasia. Annals of Neurology. 2001;49(4):425-432.

69. Roman G, Tatemichi T, Erkinjuntti T, Cummings J, Masdeu J, Garcia J et al. Vascular dementia: Diagnostic criteria for research studies: Report of the NINDS-AIREN International Workshop. Neurology. 1993;43(2):250-250.

70. Roche J, Rojas-Garcia R, Scott K, Scotton W, Ellis C, Burman R et al. A proposed staging system for amyotrophic lateral sclerosis. Brain. 2012;135(3):847-852.

71. Hoehn M, Yahr M. Parkinsonism: onset, progression, and mortality. Neurology. 1967;17(5):427-427.

72. Sheehan D, Lecrubier Y, Sheehan K, Amorin P, Janvas J, Weiler E et al. The Mini-International Neuropsychiatric Interview (M.I.N.I.): the development and validation of a structured diagnostic psychiatric interview for DSM-IV and ICD-10. Journal of Clinical Psychiatry. 1998;Suppl 20:22-33:34-57.

73. Guy, W. (1976) Clinical Global Impressions, ECDEU Assessment Manual for Psychopharmacology, revised (DHEW Publ. No. ADM 76-338). National Institute of Mental Health, Rockville, 218-222.

74. Kroenke K, Spitzer R, Williams J. The PHQ-9. Journal of General Internal Medicine. 2001;16(9):606-613.

75. Rush A, Carmody T, Reimitz P. The Inventory of Depressive Symptomatology (IDS): Clinician (IDS-C) and Self-Report (IDS-SR) ratings of depressive symptoms. International Journal of Methods in Psychiatric Research. 2000;9(2):45-59.

76. Suárez-González A, Cassani A, Gopalan R, Stott J, Savage S. When it is not primary progressive aphasia: A scoping review of spoken language impairment in other neurodegenerative dementias. Alzheimer's & Dementia: Translational Research & Clinical Interventions. 2021;7(1).

77. Bora E, Harrison B, Yücel M, Pantelis C. Cognitive impairment in euthymic major depressive disorder: a meta-analysis. Psychological Medicine. 2012;43(10):2017-2026.

78. Torres, I J et al. "Neuropsychological functioning in euthymic bipolar disorder: a meta-analysis." *Acta psychiatrica Scandinavica. Supplementum* ,434 (2007): 17-26. doi:10.1111/j.1600-0447.2007.01055.x

79. Bourne C, Aydemir Ö, Balanzá-Martínez V, Bora E, Brissos S, Cavanagh J et al. Neuropsychological testing of cognitive impairment in euthymic bipolar disorder: an individual patient data meta-analysis. Acta Psychiatrica Scandinavica. 2013;128(3):149-162.

80. Besser L, Litvan I, Monsell S, Mock C, Weintraub S, Zhou X et al. Mild cognitive impairment in Parkinson's disease versus Alzheimer's disease. Parkinsonism & Related Disorders. 2016;27:54-60.

For peer review only - http://bmjopen.bmj.com/site/about/guidelines.xhtml

81. Semkovska M, Quinlivan L, O'Grady T, Johnson R, Collins A, O'Connor J et al. Cognitive function following a major depressive episode: a systematic review and meta-analysis. The Lancet Psychiatry. 2019;6(10):851-861.

82. Kudlicka A, Clare L, Hindle J. Executive functions in Parkinson's disease: Systematic review and meta-analysis. Movement Disorders. 2011;26(13):2305-2315.

83. Gurnani A, Gavett B. The Differential Effects of Alzheimer's Disease and Lewy Body Pathology on Cognitive Performance: a Meta-analysis. Neuropsychology Review. 2016;27(1):1-17.

84. Beeldman E, Raaphorst J, Klein Twennaar M, de Visser M, Schmand B, de Haan R. The cognitive profile of ALS: a systematic review and meta-analysis update. Journal of Neurology, Neurosurgery & Psychiatry. 2015;87(6):611-619.

85. Raucher-Chéné D, Achim A, Kaladjian A, Besche-Richard C. Verbal fluency in bipolar disorders: A systematic review and meta-analysis. Journal of Affective Disorders. 2017;207:359-366.

86. Bauer K, Malek-Ahmadi M. Meta-analysis of Controlled Oral Word Association Test (COWAT) FAS performance in amnestic mild cognitive impairment and cognitively unimpaired older adults. Applied Neuropsychology: Adult. 2021;:1-7.

87. Piatt A, Fields J, Paolo A, Tröster A. Action (verb naming) fluency as an executive function measure: convergent and divergent evidence of validity. Neuropsychologia. 1999;37(13):1499-1503.

88. Beber B, Chaves M. The Basis and Applications of the Action Fluency and Action Naming Tasks. Dementia & Neuropsychologia. 2014;8(1):47-57.

89. Ramos A, Machado L. A Comprehensive Meta-analysis on Short-term and Working Memory Dysfunction in Parkinson’s Disease. Neuropsychology Review. 2021;31(2):288-311.

90. Rabi R, Vasquez B, Alain C, Hasher L, Belleville S, Anderson N. Inhibitory Control Deficits in Individuals with Amnestic Mild Cognitive Impairment: A Meta-Analysis. Neuropsychology Review. 2020;30(1):97-125.

91. Clarke N, Barrick T, Garrard P. A Comparison of Connected Speech Tasks for Detecting Early Alzheimer’s Disease and Mild Cognitive Impairment Using Natural Language Processing and Machine Learning. Frontiers in Computer Science. 2021;3.

92. Goodglass, H. and E. Kaplan. 1983. The Assessment of Aphasia and Related Disorders, ed. 2. Lea and Febiger, Philadelphia, PA.

93. Nicholas L, Brookshire R. A System for Quantifying the Informativeness and Efficiency of the Connected Speech of Adults with Aphasia. Journal of Speech, Language, and Hearing Research. 1993;36(2):338-350.

94. Teh E, Yap M, Liow S. PiSCES: Pictures with social context and emotional scenes with norms for emotional valence, intensity, and social engagement. Behavior Research Methods. 2017;50(5):1793-1805.

For peer review only

95. Sherratt S, Bryan K. Textual cohesion in oral narrative and procedural discourse: the effects of ageing and cognitive skills. International Journal of Language & Communication Disorders. 2018;54(1):95-109.

96. Deterding D. The North Wind versus a Wolf: short texts for the description and measurement of English pronunciation. Journal of the International Phonetic Association. 2006;36(2):187-196.

97. Rusz J, Cmejla R, Tykalova T, Ruzickova H, Klempir J, Majerova V et al. Imprecise vowel articulation as a potential early marker of Parkinson's disease: Effect of speaking task. The Journal of the Acoustical Society of America. 2013;134(3):2171-2181.

98. Rong P, Yunusova Y, Richburg B, Green J. Automatic extraction of abnormal lip movement features from the alternating motion rate task in amyotrophic lateral sclerosis. International Journal of Speech-Language Pathology. 2018;20(6):610-623.

99. Spitzer R, Kroenke K, Williams J, Löwe B. A Brief Measure for Assessing Generalized Anxiety Disorder. Archives of Internal Medicine. 2006;166(10):1092.

100. Moulton C, Strawbridge R, Tsapekos D, Oprea E, Carter B, Hayes C et al. The Maudsley 3-item Visual Analogue Scale (M3VAS): Validation of a scale measuring core symptoms of depression. Journal of Affective Disorders. 2021;282:280-283

101. Altman E, Hedeker D, Peterson J, Davis J. The Altman Self-Rating Mania Scale. Biological Psychiatry. 1997;42(10):948-955.

102. Lenain R, Weston J, Shivkumar A, Fristed E. Surfboard: Audio Feature Extraction for Modern Machine Learning [Internet]. arxiv.org. 2020 [cited 12 April 2022]. Available from: https://arxiv.org/abs/2005.08848

103. Weston, J., Lenain, R., Meepegama, U. & Fristed, E.. (2021). Learning de-identified representations of prosody from raw audio. Proceedings of the 38th International Conference on Machine Learning, in: Proceedings of Machine Learning Research. 139:11134-11145 Available from https://proceedings.mlr.press/v139/weston21a.html.

104. Qi P, Zhang Y, Zhang Y, Bolton J, Manning C. Stanza : A Python Natural Language Processing Toolkit for Many Human Languages [Internet]. 2003 [cited 12 April 2022]. Available from: https://arxiv.org/pdf/2003.07082.pdf

105. Pedregosa F, Varoquaux G, Gramfort A, Michel V, Thirion B, Grisel O et al. Scikit-learn: Machine Learning in Python [Internet]. 2011 [cited 12 April 2022]. Available from: https://jmlr.csail.mit.edu/papers/volume12/pedregosa11a/pedregosa11a.pdf

106. Weston J, Lenain R, Meepegama U, Fristed E. https://arxiv.org/pdf/2107.08251.pdf [Internet]. 2022 [cited 12 April 2022]. Available from: https://arxiv.org/pdf/2107.08251.pdf

107. Mann H, Whitney D. On a Test of Whether one of Two Random Variables is Stochastically Larger than the Other. The Annals of Mathematical Statistics. 1947;18(1):50-60.

108. Cooper D, Epker M, Lacritz L, Weiner M, Rosenberg R, Honig L et al. Effects of Practice on Category Fluency in Alzheimers Disease*. The Clinical Neuropsychologist. 2001;15(1):125-128.

For peer review only

For peer review only - http://bmjopen.bmj.com/site/about/guidelines.xhtml

109. Cooper D, Lacritz L, Weiner M, Rosenberg R, Cullum C. Category Fluency in Mild Cognitive Impairment. Alzheimer Disease & Associated Disorders. 2004;18(3):120-122.

FIGURE TITLE

Figure 1. Flow diagram of the participant journey in the Rhapsody study

For peer review only

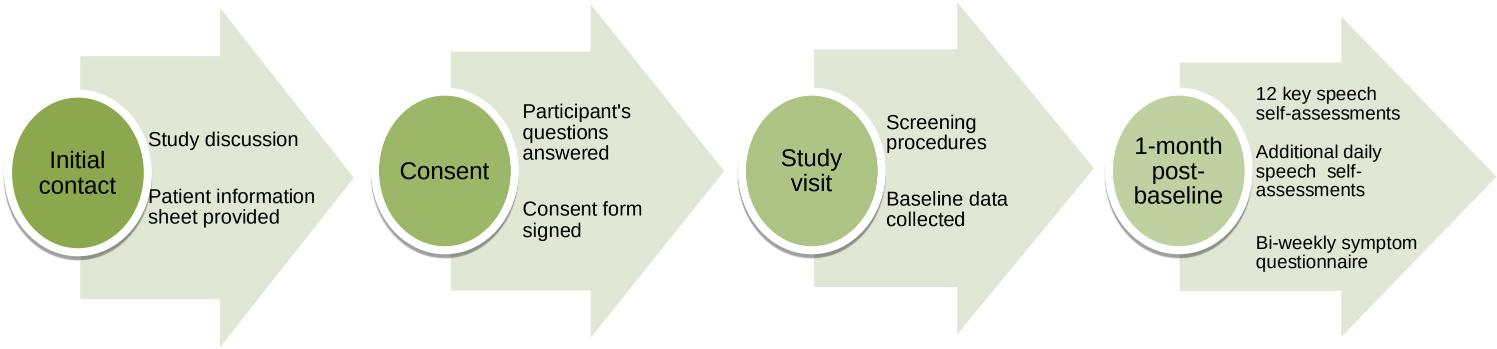

Figure 1.

For peer review only
